# Isolation and Structures of Polyarene Palladium Nanoclusters

**DOI:** 10.1021/jacs.3c02849

**Published:** 2023-06-05

**Authors:** Ayaka Hatano, Tsuyoshi Sugawa, Rei Mimura, Shunichi Kataoka, Koji Yamamoto, Tsubasa Omoda, Bo Zhu, Yu Tian, Shigeyoshi Sakaki, Tetsuro Murahashi

**Affiliations:** † Department of Chemical Science and Engineering, School of Materials and Chemical Technology, Tokyo Institute of Technology, O-okayama, Meguro-ku, Tokyo 152-8552, Japan; ‡ Institute for Integrated Cell-Material Sciences (iCeMS), Kyoto University, Sakyo-ku, Kyoto 606-8302, Japan

## Abstract

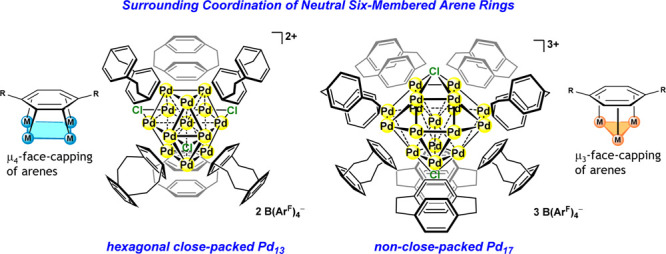

We report that surrounding coordination of neutral six-membered arene rings affords molecularly well-defined organotransition metal nanoclusters. With the use of [2.2]paracyclophane as the face-capping arene ligand, we have isolated two polyarene palladium nanoclusters, one consisting of a hexakis-arene ligand shell and a hexagonal close-packed Pd_13_ anticuboctahedron trichloride core, and the other consisting of an octakis-arene ligand shell and a non-close-packed Pd_17_ square gyrobicupola dichloride core, both with Pd–Pd direct bonding. The μ_4_-facial coordination mode of arene was discovered through the structural characterization of the Pd_13_ cluster. Their Pd_13_ and Pd_17_ cores, which are distinct from the previously identified face-centered-cubic Pd_13_ core surrounded by seven-membered cycloheptatrienyl, are explained by stereochemical and theoretical analyses.

The size and packing-selective construction of molecular metal nanoclusters has been a longstanding challenge in inorganic chemistry, organometallic chemistry, catalysis, and materials science.^1−4^ For example, the 13-atom clusters have been thought to provide good models for studying metal nanoclusters because they possibly adopt three compact and symmetrical structures composed of 12 surface atoms and one interstitial atom, where each corresponds to face-centered-cubic (*fcc*) close-packed cuboctahedron, hexagonal close-packed (*hcp*) anticuboctahedron, and non-close-packed icosahedron.^5−8^ However, it is usually difficult to deduce the 13-atom metal core structure from its ligand shell due to the presence of many ligands for covering the surface sites.^9−11^ Recently, our group found that the use of C_7_H_7_ (Tr), a seven-membered cyclic unsaturated hydrocarbon (CUH), as the face-capping ligands provides an *fcc*-close-packed cuboctahedral 13-atom cluster [Pd_13_Tr_6_]^2+^ selectively.^12^ In the Pd_13_ cluster, only six Tr ligands surround the 13-atom metal core, giving a simple octahedral ligand geometry lying within the stereochemical regime developed for 6-coordinate mononuclear complexes. In order to verify the usefulness of this face-capping strategy using CUH ligands, it is necessary to understand whether and how the coordination of face-capping ligands affect the size and packing pattern of the metal core.^13,14^ Here we isolated and characterized transition metal nanoclusters surrounded by neutral six-membered arene rings (Figure 1). The distinct molecular structures of the polyarene nanoclusters, a 13-atom *hcp*-anticuboctahedral Pd cluster surrounded by six arene and three chloride ligands and a 17-atom non-close-packed square gyrobicupola Pd cluster surrounded by eight arene and two chloride ligands, were elucidated and characterized based on the experimental and theoretical analyses.

**Figure 1:**
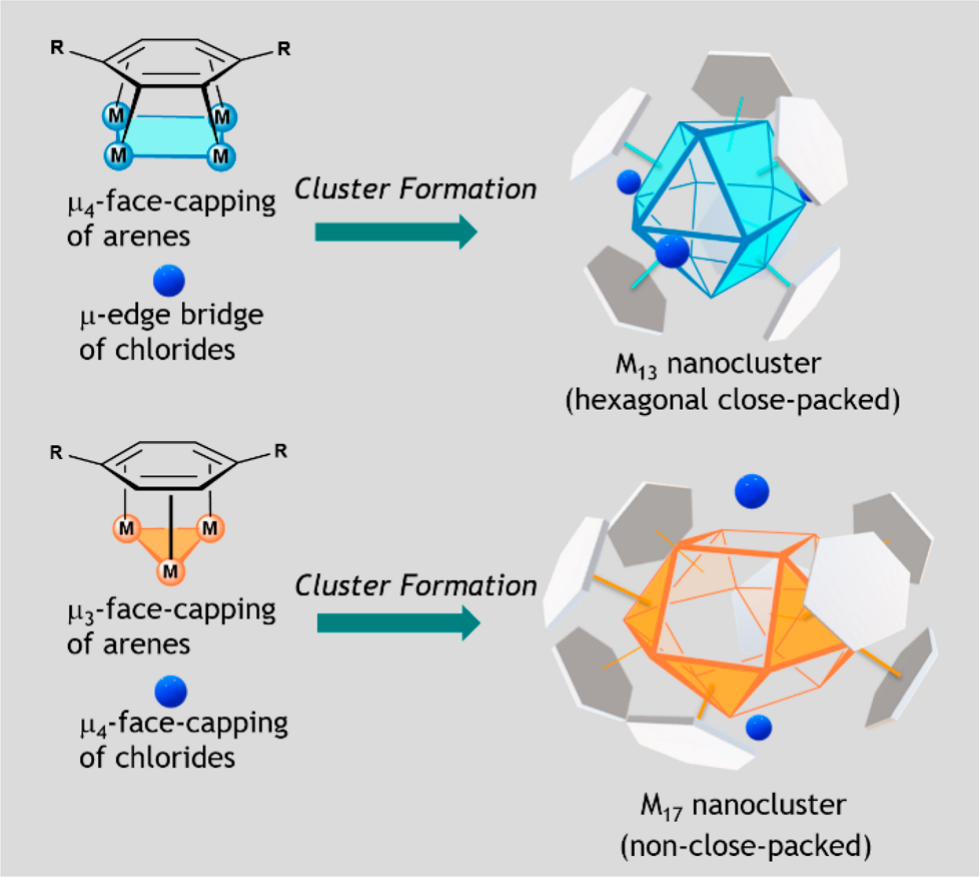
Facial coordination of six-membered ring C_6_H_4_R_2_ to square faces gives hexagonal close-packed 13-atom anticuboctahedron, and that to triangular faces gives non-close-packed 17-atom square gyrobicupola, where each is further capped by chloride ligands (blue balls).

Although π-coordination of neutral six-membered arene rings has been widely used in organometallic chemistry,^15,16^ the arene π-coordination has not led to molecularly well-defined metal nanoclusters which contain more than 10 metal atoms. For example, the self-assembly of half-sandwich η^6^-arene mononuclear metal moieties has only provided small metal clusters containing up to four metal atoms.^17^ Although μ_3_-face-capping coordination of arenes has previously been identified in small M_*m*_ clusters (*m* ≤ 6),^18−20^ it is challenging to surround a metal nanocluster core by using multiple μ_3_-arene ligands to construct polyarene metal nanoclusters. Moreover, the *fcc*- and *hcp*-M_13_ cores have tetranuclear faces in addition to trinuclear faces, but it is not clear whether a six-membered arene ring has the ability to face-cap a tetranuclear face through μ_4_-coordination. The possible existence of polyarene metal nanoclusters containing up to 10 metal atoms was discussed previously on the basis of the mass spectrometric detection of M_*m*_(benzene)_*n*_ species (M = Fe, Co, Ni; *m* > *n*, *m* ≈ 10, *n* ≈ 6) in gas-phase reactions of metal vapor with benzene.^21^ However, such compounds have not been isolated and their atomic structures remain unexplained.

We selected [2.2]paracyclophane (PCP) as the arene ligand in our study because it shows a greater coordination ability than benzene due, in part, to transannular electronic interactions between the two parallel arene rings.^22,23^ Our group previously reported that the bis-μ_3_-arene trinuclear sandwich complex [Pd_3_(μ_3_-PCP)_2_(CH_3_CN)_3_][B(Ar^F^)_4_]_2_ (**1**) [Ar^F^ = 3,5-(F_3_C)_2_C_6_H_3_] is isolable although the corresponding bis-benzene Pd_3_ complexes could not be obtained.^24,25^ Interestingly, ^1^H NMR monitoring of a solution of **1** in 1,2-dichloroethane-*d*_4_ (DCE-*d*_4_) at 70 °C showed a gradual consumption of **1**, accompanied by the generation of two products exhibiting a large downfield shift for one of the two methylene proton resonances (δ = 20.00; δ = 9.74) and a large upfield shift for one of the two phenylene resonances (δ = −6.26; δ = −6.28), characteristic of paramagnetic compounds. Heating **1** in DCE for 1 day gave a 46% yield of a mixture of two products in a molar ratio of 56:44. Separation of the products by precipitation from DCE–Et_2_O gave a hexakis-PCP Pd_13_ cluster, [Pd_13_(μ_4_-PCP)_6_(μ-Cl)_3_][B(Ar^F^)_4_]_2_ (**2**), in 15% yield, whereas an octakis-PCP Pd_17_ cluster, [Pd_17_(μ_3_-PCP)_8_(μ_4_-Cl)_2_][B(Ar^F^)_4_]_3_ (**3**), was obtained in 8% yield by crystallization from the black supernatant (Figure 2a). It is reasonable to assume that B(Ar^F^)_4_^–^ behaves as a mild reducing reagent in the production of **2** and **3** in solution,^26^ as the formation of biaryl^27^ and CH_3_CN–B(Ar^F^)_3_^28^ were detected during the synthesis.^29^ The Cl ligands in **2** and **3** might be abstracted from the DCE solvent.

**Figure 2:**
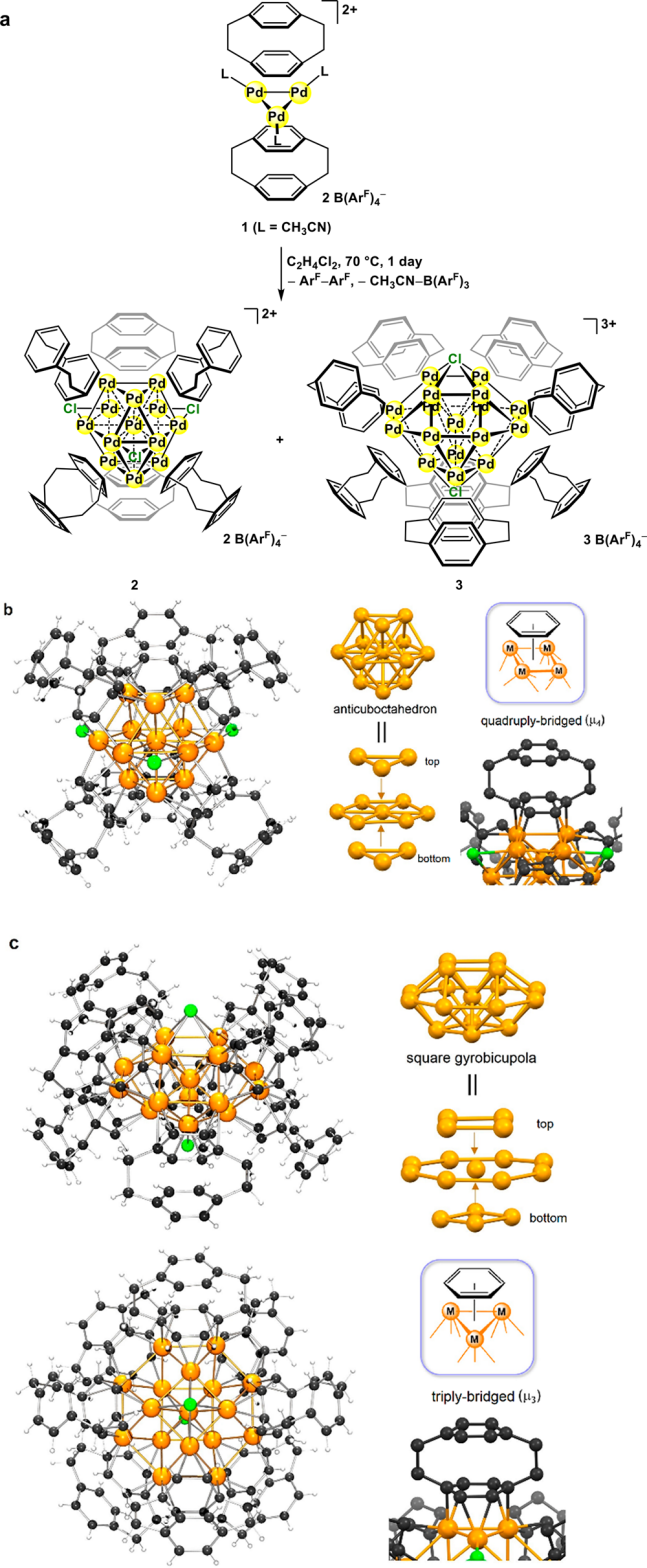
(a) Synthesis of [Pd_13_(μ_4_-PCP)_6_(μ-Cl)_3_][B(Ar^F^)_4_]_2_ (**2**) and [Pd_17_(μ_3_-PCP)_8_(μ_4_-Cl)_2_][B(Ar^F^)_4_]_3_ (**3**). (b) Ball-and-stick representation of the X-ray structure of **2**, a drawing of the anticuboctahedral Pd_13_ core of **2**, and a drawing highlighting the μ_4_-coordination mode of the PCP ligands in **2**. (c) Ball-and-stick representation of the X-ray structure of **3**, a drawing of the square gyrobicupola Pd_17_ core of **3**, and a drawing highlighting the μ_3_-coordination mode of the PCP ligands in **3**. For (b) and (c), orange = Pd; green = Cl; dark gray = C; the counteranions and solvent molecules were omitted for clarity.

The structures of **2** and **3** were determined by single-crystal X-ray diffraction analysis. Nanocluster **2** contains an *hcp*-Pd_13_ core with an anticuboctahedral Pd_12_ surface arrangement (Johnson solid J_27_) and one interstitial Pd atom (Figure 2b). Each PCP ligand facially caps a Pd_4_ face of the Pd_13_ core through μ_4_-bridging coordination, which is an unprecedented coordination mode for six-membered arene rings. The observation of this coordination mode in **2** may result from the unique properties of the nanosized metal cluster core that are lacking in smaller M_*n*_ complexes (e.g., *n* = 4). Coverage of the six square Pd_4_ faces of the anticuboctahedral Pd_13_ core results in a triangular prismatic arrangement of the PCP ligands, providing additional spaces at the equatorial edges to accommodate three μ-Cl ligands. As a result, six PCP and three Cl ligands form a tricapped triangular prismatic ligand geometry with a symmetric (pseudo-*D*_3*h*_) structure. The Pd–Pd bond lengths in the Pd_13_ core of **2** [2.5392(9)–2.7837(6) Å] are in the normal range for Pd–Pd bond lengths (cf. 2.75 Å in bulk Pd)^30^ except for the elongated chloro-bridged Pd–Pd bonds [3.0167(6), 3.0146(8), and 3.0146(8) Å]. Although the anticuboctahedral M_13_ core is one of the basic close-packed geometries for metal nanoclusters, it has been rarely obtained except for the rhodium hydride carbonyl clusters [Rh_13_H_5-*n*_(CO)_24_]^*n*−^.^9^ Notably, formation of the *hcp*-anticuboctahedral Pd_13_ core with the use of arenes as the face-capping ligands is in sharp contrast to that of *fcc*-cuboctahedral Pd_13_ core in [Pd_13_Tr_6_]^2+^ where C_7_H_7_ ligands are employed as the face-capping ligands.^12^

The Pd_17_ core in **3** has a non-close-packed, square gyrobicupola geometry (Johnson solid J_29_) with one interstitial Pd atom (Figure 2c). The eight PCP ligands surround the Pd_17_ core in a square antiprismatic geometry, and each PCP ligand facially caps a triangular Pd_3_ face of the square gyrobicupola through μ_3_-bridging coordination, despite the presence of eight square Pd_4_ faces at the surface. The eight PCP and two Cl ligands adopt a bicapped square antiprismatic geometry with a pseudo-*D*_4*d*_ symmetry. The Pd–Pd bond lengths [2.5649(4)–2.8937(5) Å] are in the normal range for Pd–Pd bonds,^30^ and the interstitial Pd atom is bonded to the eight Pd atoms at the square faces capped by Cl ligands but not to the Pd atoms forming the eight-membered equatorial ring (Pd···Pd ≥ 3.59 Å). The 17-atom square gyrobicupola nanocluster is unprecedented. It is noticeable that the icosahedral core, a more common non-close-packed M_13_ structure for group 11 metals,^3,4^ was not observed in the present case.

The different Pd packing in the 13-atom clusters **2** and [Pd_13_Tr_6_]^2+^ can be understood qualitatively by considering coordination numbers, valence electron numbers, and geometrical adaptation between the metal core and the ligand shell, where each is dependent on the ring-size of the facial CUH ligands. The difference of the coordination numbers between **2** and [Pd_13_Tr_6_]^2+^ results from the presence or absence of Cl ligands. According to the empirical or semiempirical approach,^1^ the M_13_ cluster in either cuboctahedron or anticuboctahedron would prefer 170 valence electrons. Although [Pd_13_Tr_6_]^2+^ is exactly the 170e compound and thus coordinatively saturated, [Pd_13_(μ_4_-arene)_6_]^2+^ which lacks Cl ligands might be highly electron-deficient (164e) because the number of electrons donated by a ligand is reduced by one when changing the ligands from seven-membered C_7_H_7_ to six-membered arene rings. Therefore, the acceptance of three μ-Cl ligands might well mitigate the electron-deficient state of the cluster.

From a stereochemical viewpoint, an *hcp*-anticuboctahedron has three uncoordinated edges after μ_4_-facial coordination of six CUH ligands, providing suitable sites for accommodation of μ-Cl ligands (Figure 3). On the other hand, it is unlikely that the *fcc*-cuboctahedron metal core possessing six face-capping CUH ligands accommodates additional Cl ligands, because all M–M edges are involved in the facial coordination of CUH ligands. It is noted that the observed metal core geometry correlates with the ideal ligand geometry expected from the ligand number. For **2**, the square face augmentation^31^ of the anticuboctahedral core leads to the trigonal prismatic geometry of six face-capping ligands, and additional edge augmentation at the three uncoordinated equatorial edges gives a tricapped trigonal prism geometry, which is an ideal ligand geometry for 9-coordinate complexes (Figure S20).^32^ The case of [Pd_13_Tr_6_]^2+^ is simpler; square face augmentation of cuboctahedron produces octahedron, an ideal 6-coordinate ligand geometry.

**Figure 3:**
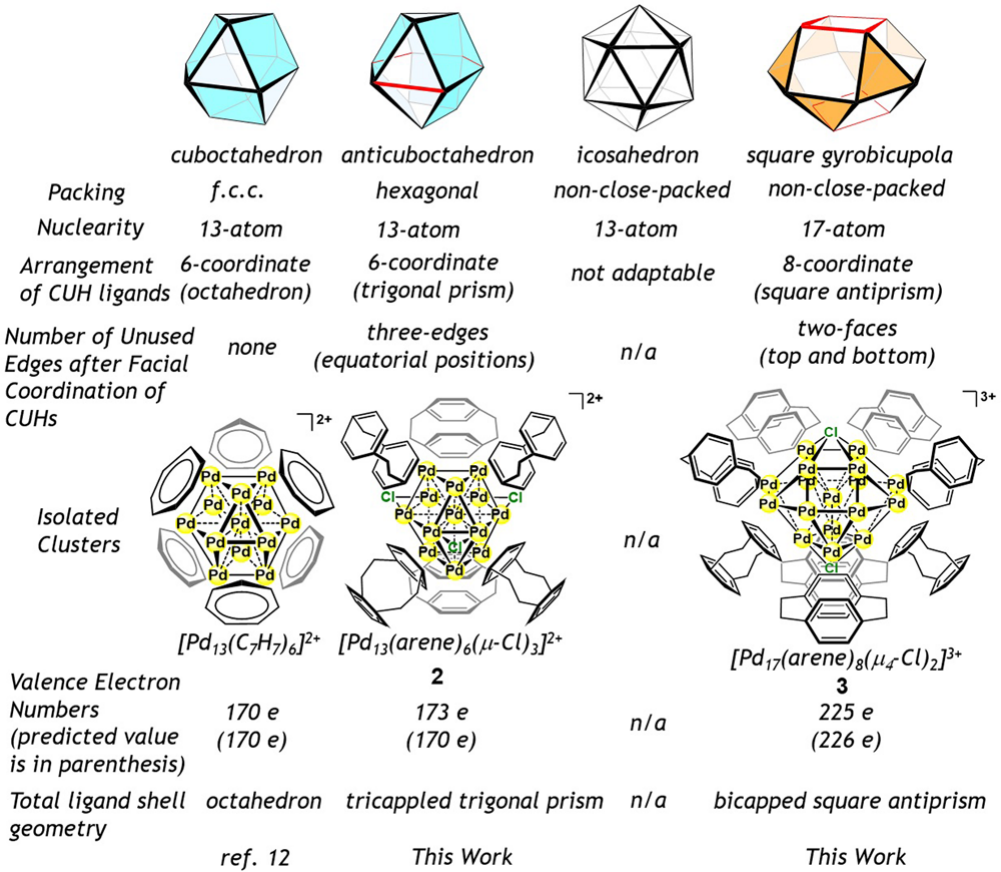
Summary of the four metal core structures: cuboctahedron, anticuboctahedron, icosahedron, and square gyrobicupola. The square faces or the triangular faces used for face-capping coordination of CUH ligands are shown in sky blue or orange. The edges which are not involved in the facial coordination of CUHs are shown in red.

Such consideration also explains the fact that the non-close-packed square gyrobicupola Pd_17_ cluster **3** is favorably formed, while the non-close-packed icosahedron Pd_13_ cluster is not. The number of valence electrons of **3** (225e, where μ_4_-Cl is considered as a five-electron-donor ligand) is close to that for an M_17_ cluster (226e) which is predicted by an empirical approach (Figure 3), where the accommodation of two μ_4_-Cl ligands mitigates the electron-deficiency of [Pd_17_(μ_3_-arene)_8_]^3+^ (215e). The μ_4_-coordination of arenes is not compatible with the μ_4_-Cl capping because the top and bottom Pd_4_ squares are used for the arene binding. A bicapped square antiprism geometry is known as an ideal ligand geometry for 10-coordinate metal complexes,^32^ to which the observed square gyrobicupola Pd_17_ core is geometrically adaptable through the triangular face augmentation with eight arenes and additional square-face augmentation with two Cl ligands (Figure S20). The non-close-packed icosahedral metal core would be unlikely to be formed when using face-capping CUH ligands, because the ligand geometry generated by face augmentation from icosahedron seems not to match any ideal coordination geometry of ligands.

In order to gain further insights into the present polyarene metal clusters, we carried out density functional theory (DFT) calculations on [Pd_13_(μ_4_-PCP)_6_(μ-Cl)_3_]^2+^ (**2**^**2+**^), [Pd_13_(μ_4_-PCP)_6_(μ-Cl)_3_]^+^ (**2**^**+**^), [Pd_17_(μ_3_-PCP)_8_(μ_4_-Cl)_2_]^3+^ (**3**^**3+**^), and [Pd_17_(μ_3_-PCP)_8_(μ_4_-Cl)_2_]^4+^ (**3**^**4+**^). As shown in Figure 4a, the HOMO of **2**^**+**^ mainly involves 4d_σ_–4d_σ_ antibonding interactions in the top and bottom Pd_3_ moieties (these Pd_3_ moieties are defined in Figure 2b). Three MOs mainly composed of Pd 4d orbitals are found in unoccupied level (LUMO to LUMO+2), whereas a [Pd_13_]^4+^ core would have two MOs mainly composed of Pd 4d orbitals in the unoccupied level. This feature is not unreasonable at all because the 5s orbital at the interstitial Pd atom becomes occupied through Pd 4d–5s hybridization (Figure S19) and instead one MO mainly composed of Pd 4d becomes unoccupied (the LUMO). This LUMO involves the 4d_π_–3p_π_ antibonding interaction between the equatorial Pd atoms and μ-Cl ligands, that is, the origin of the relatively short Pd–Pd bonds (2.5392(9) Å, 2.5477(6) Å) at the unbridged (no μ-Cl) equatorial positions. The degenerated LUMO+1 and LUMO+2 are featured by the antibonding interaction between the arene π-orbitals and surface Pd 4d orbitals (Figure 4b, left-hand side). These MOs are indeed reminiscent of the e″ orbitals of the 6-coordinate trigonal prismatic mononuclear complexes,^33^ and their vacancy suggests that donation occurs from the CUH ligands to the surface Pd atoms. In **3**^**4+**^, the LUMO and LUMO+1 are mainly featured by the σ-type antibonding interactions between the top and bottom Pd_4_ squares and equatorial Pd_8_ ring and also by the antibonding interaction between the arene π-orbitals and the surface Pd d-orbitals (Figure 4b, right-hand side). The HOMO involves the arene–Pd antibonding interaction, whereas the 4d_σ_–4d_σ_ antibonding character is rather small. The 4d–5s hybridization occurs at the interstitial Pd atom as found in **2**^**+**^. The Hirshfeld charges of the metal cores in **2**^**2+**^ and **3**^**3+**^ are similar; +1.507*e* and +1.579 *e*, respectively, despite the different numbers of Pd atoms, PCP ligands, and Cl ligands in addition to the different total positive charge (Table S6). DFT calculations also indicated that the accommodation of three Cl ligands in **2**^**2+**^ or two Cl ligands in **3**^**3+**^ from DCE is thermodynamically favorable (Table S7).

**Figure 4:**
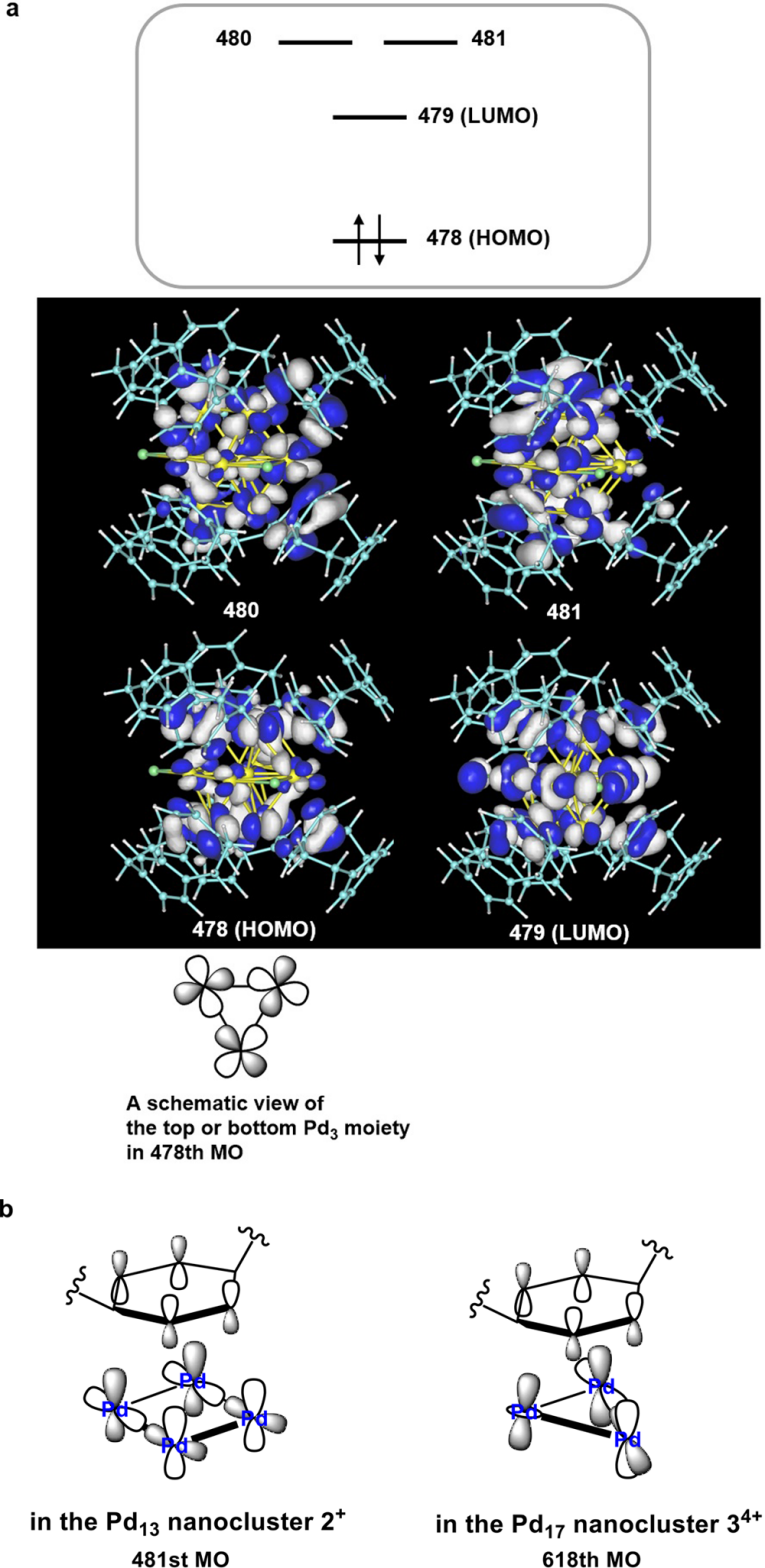
(a) Frontier molecular orbitals of [Pd_13_(μ_4_-PCP)_6_(μ-Cl)_3_]^+^ (**2**^**+**^). (b) Antibonding interaction between the arene π-orbitals and Pd_3_ or Pd_4_ d-orbitals.

Cyclic voltammetry (CV) analyses of **2** and **3** indicated that there are several readily addressable oxidation states in which the cluster frameworks are retained: + 0.36 V (Δ*E*_p_ = 68 mV) and −0.30 V (Δ*E*_p_ = 66 mV) for **2**; +0.83 V (Δ*E*_p_ = 71 mV), +0.20 V (Δ*E*_p_ = 68 mV), and −0.35 V (Δ*E*_p_ = 65 mV) for **3** (Figure 5). A one-electron reduction product [Pd_13_(μ_4_-PCP)_6_(μ-Cl)_3_][B(Ar^F^)_4_] (**2′**) from **2** and a one-electron oxidation product [Pd_17_(μ_3_-PCP)_8_(μ_4_-Cl)_2_][B(Ar^F^)_4_]_4_ (**3′**) from **3** were generated in CD_2_Cl_2_ by addition of Et_3_N or AgPF_6_, respectively; each product showed ^1^H NMR resonances in the normal chemical-shift region for diamagnetic compounds. A single set of four resonances for the PCP ligands was observed in the ^1^H and ^13^C NMR spectra of **2′** and **3′** at 20 °C in CD_2_Cl_2_, and no apparent changes in their resonance patterns were observed, even at −90 °C (although a broadening of the resonance for the coordinated arene protons was observed), indicating that each PCP ligand rotates fluxionally on the NMR time scale in solution. The molecular structure of **2′** was determined by X-ray diffraction analysis, and its structure was found to be quite similar to that of **2** (Figure S15).

**Figure 5:**
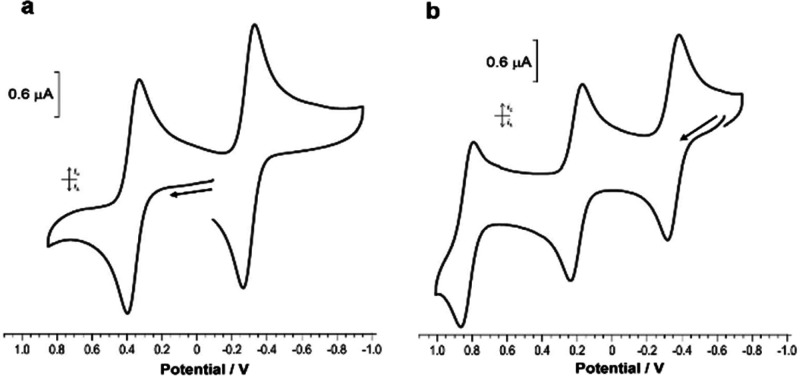
(a) Cyclic voltammogram of **2**. (b) Cyclic voltammogram of **3**. For (a) and (b), a solution of the cluster (0.5 mM) in CH_2_Cl_2_ with [*n*-Bu_4_N][B(Ar^F^)_4_] (0.1 M) was analyzed at a scan rate of 100 mV/s. The reference potential is that of the Fe(C_5_H_5_)_2_/[Fe(C_5_H_5_)_2_]^+^ redox couple.

In conclusion, we synthesized and structurally characterized the polyarene metal nanoclusters. It has been proven that the face-capping coordination of neutral six-membered arene rings well stabilizes symmetrical metal nanocluster cores. The μ_4_-coordination of arene was also discovered in metal nanoclusters. The analysis of the present organotransition metal nanoclusters suggested that the ring-size of facially coordinated CUH ligands has a pronounced impact on the structures of metal nanoclusters through steric and electronic perturbations.

## Supporting Information Available

Full experimental details, including synthetic procedures and characterization details, cyclic voltammetry, crystallographic and computational details (PDF)
