# Supplementary material for: Isolation and Structures of Polyarene Palladium Nanoclusters
Source: J Am Chem Soc. 2023 Jun 5;145(28):15030–5. doi: 10.1021/jacs.3c02849 (PMC10360153; doi:10.1021/jacs.3c02849)
Supplement: Supplementary file 1 — ja3c02849_si_001.pdf [file ja3c02849_si_001.pdf]

Supporting Information

Isolation and Structures of Polyarene Palladium Nanoclusters

Ayaka Hatano,[1] Tsuyoshi Sugawa,[1] Rei Mimura,[1] Shunichi Kataoka,[1] Koji Yamamoto,[1] Tsubasa Omoda,[1] Bo Zhu,[2] Yu Tian,[2] Shigeyoshi Sakaki[2,*] and Tetsuro Murahashi[1,*]

[1]*Department of Chemical Science and Engineering, School of Materials and Chemical Technology, Tokyo Institute of Technology, O-okayama, Meguro-ku, Tokyo 152-8552, Japan*

[2]*Institute for Integrated Cell-Material Sciences (iCeMS), Kyoto University, Sakyo-ku, Kyoto 606-8302, Japan*

Correspondence to: murahashi.t.aa@m.titech.ac.jp
sakaki.shigeyoshi.47e@st.kyoto-u.ac.jp

Table of Contents

Supplementary Methods	3
General Methods	3
Synthesis of $[Pd_{13}(\mu_4\text{-PCP})_6(\mu\text{-Cl})_3][B(Ar^F)_4]_2$ (**2**)	4
Synthesis of $[Pd_{17}(\mu_3\text{-PCP})_8(\mu_4\text{-Cl})_2][B(Ar^F)_4]_3$ (**3**)	7
Synthesis of $[Pd_{13}(\mu_4\text{-PCP})_6(\mu\text{-Cl})_3][B(Ar^F)_4]$ (**2'**)	11
Synthesis of $[Pd_{17}(PCP)_8Cl_2][BAr^F{}_4]_4$ (**3'**)	14
Synthesis of $[Pd_3(\mu_3\text{-PCP})_2(PhCN)_3][PF_6]_2$ (**1'**)	17
Electrochemical Measurements	18
X-ray Crystallographic Analyses	21
X-ray Crystallographic Data	22
Computational Details	30
Stereochemical Correlation of the Metal Cluster Core and the Ligand Shell	63
Supplementary References	64

Supplementary Methods

General methods

All manipulations involving air- and moisture-sensitive compounds were conducted under a nitrogen atmosphere using standard Schlenk or dry-box technique. ^{1}H and $^{13}C\{^{1}H\}$ NMR spectra were recorded on 400 MHz instruments (JEOL JNM-ECZ400S). The chemical shifts were referenced to the residual resonances of deuterated solvents. Elemental analyses were performed on a PerkinElmer 2400II series CHN analyzer. X-ray crystal data were collected by Rigaku XtaLAB Synergy diffractometer equipped with a HyPix-6000HE Hybrid Photon Counting (HPC) detector and dual Mo and Cu microfocus sealed X-ray source. ESI-MS spectra were recorded on Bruker micrOTOF ESI-TOF. Cyclic voltammograms were obtained by ALS 600A electrochemical analyzer. Unless specified, all reagents were purchased from commercial suppliers and used without purification. Dichloroethane (ACS reagent grade (≥99.0%)) was purchased from Merck KGaA. Nitromethane, *n*-hexane, dichloromethane, diethyl ether, CD_3NO_2, CD_2Cl_2 and 1,2-dihchloroethane-d_4 were purified according to the standard procedures. $[Pd_3(\mu_3\text{-}PCP)_2(CH_3CN)_3][B(Ar^F)_4]_2$ (**1**) (PCP = [2.2]paracyclophane, Ar^F = 3,5-$(CF_3)_2C_6H_3$),[1] and $NaB(Ar^F)_4$,[2] were prepared according to the literature. $[Pd_2(PhCN)_6][PF_6]_2$ was prepared by ligand exchange of $[Pd_2(CH_3CN)_6][PF_6]_2$ in benzonitrile solution.[3]

Synthesis of $[Pd_{13}(\mu_4\text{-PCP})_6(\mu\text{-Cl})_3][B(Ar^F)_4]_2$ (**2**)

The suspension of $[Pd_3(\mu_3\text{-PCP})_2(CH_3CN)_3][B(Ar^F)_4]_2$ (**1**) (2.00 g, 7.74 x 10^{-1} mmol) in 1,2-dichloroethane (*ca.* 8 mL) was stirred for 1 day at 70 °C. The reaction mixture was filtered through Celite, and the filtrate was concentrated *in vacuo*. The filtrate was precipitated with Et_2O to afford a mixture of $[Pd_{13}(\mu_4\text{-PCP})_6(\mu\text{-Cl})_3][B(Ar^F)_4]_2$ (**2**) and $[Pd_{17}(\mu_3\text{-PCP})_8(\mu_4\text{-Cl})_2][B(Ar^F)_4]_3$ (**3**) (377 mg, **2**:**3** = 56:44). The mixture was dissolved in *ca.* 5 mL of 1,2-dichloroethane, and then the solution was precipitated by addition of *ca.* 10 mL of Et_2O, followed by washed with Et_2O to gave a black powder of **2** (116 mg, 2.60 x 10^{-2} mmol, 15% yield). Recrystallization from 1,2-dichloroethane/Et_2O gave black crystals. ^{1}H NMR (400 MHz, CD_2Cl_2, 5.0 mM, 25 °C) δ 19.63 (br, 24H, cyclophane CH_2), 7.74 (s, 16H, *o*-BAr^F_4), 7.57 (s, 8H, *p*-BAr^F_4), 6.92 (s, 24H, cyclophane ArH), 1.39 (br, 24H, cyclophane CH_2), -6.59 (br, 24H, cyclophane Ar*H*). ^{13}C{^{1}H} NMR (101 MHz, CD_2Cl_2, 25 °C) δ 162.1 (q, $^1J_{C\text{-}B}$ = 50 Hz, *ipso*-BAr^F), 144.4 (s, cyclophane *ipso-Ar*), 141.7 (s, cyclophane *Ar*H), 139.3 (br, cyclophane *C*H_2), 137.5 (br, cyclophane *Ar*H), 135.1 (s, *o*-BAr^F)), 129.1 (q, $^2J_{C\text{-}F}$ = 29 Hz, *m*-BAr^F), 124.9 (q, $^1J_{C\text{-}F}$ = 274 Hz, CF_3), 117.9 (m, *p*-BAr^F). Signals for one set of *ipso*-carbons and one methylene-carbons in cyclophane ligands were not observed. Anal. Calcd. For. $C_{160}H_{120}B_2Cl_3F_{48}Pd_{13}$: C, 43.03; H, 2.71. Found: C, 42.83; H, 2.50.

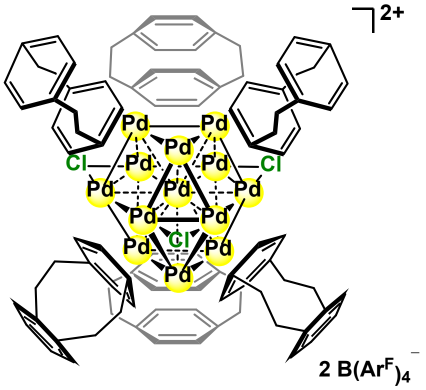

2

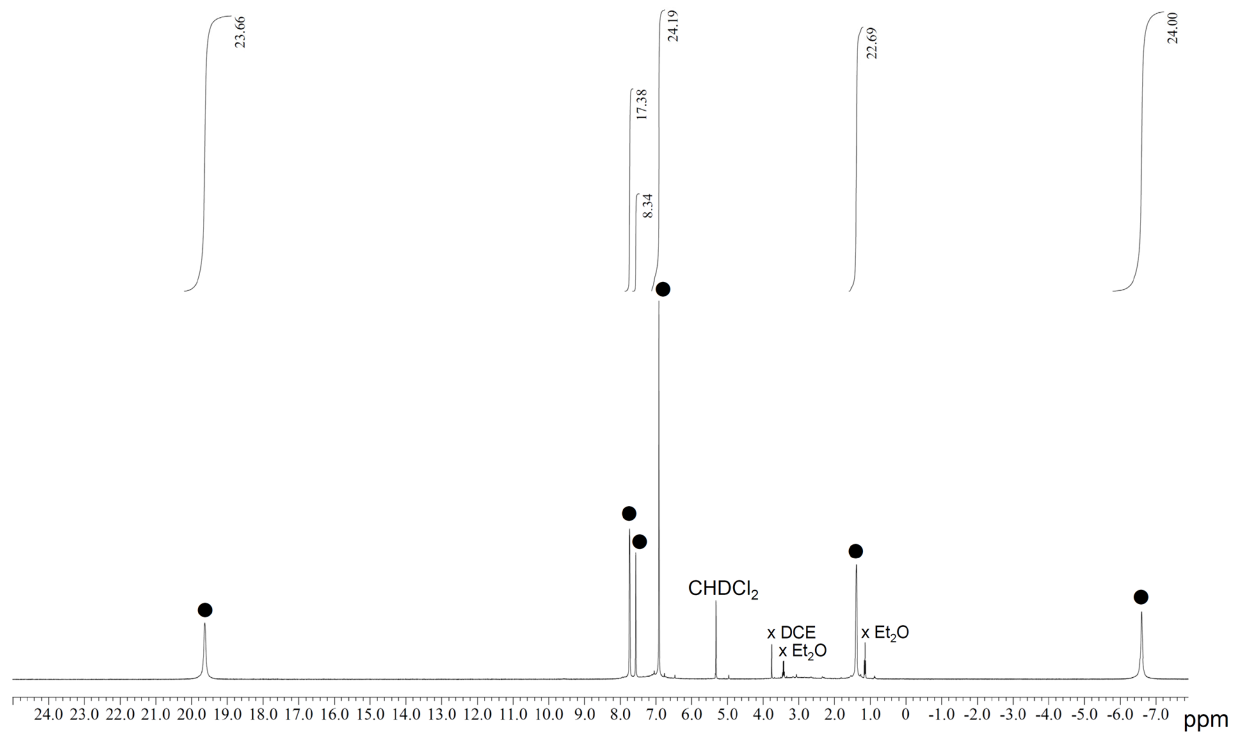

Figure S1. ^{1}H NMR spectrum of $[Pd_{13}(\mu_4\text{-PCP})_6(\mu\text{-Cl})_3][B(Ar^F)_4]_2$ (**2**). • = $[Pd_{13}(\mu_4\text{-PCP})_6(\mu\text{-Cl})_3][B(Ar^F)_4]_2$, x = residual solvent signals (Et_2O = diethyl ether, DCE = 1,2-dichloroethane).

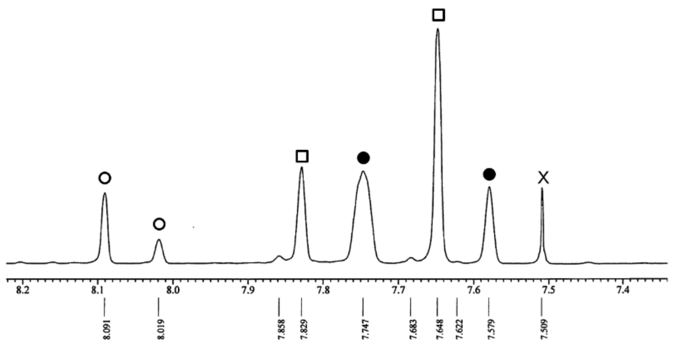

Figure S2. ^{1}H NMR spectrum of the crude sample in CD_2Cl_2, which was obtained after the reaction in DCE-d_4 for 3 h, followed by the evaporation and addition of CD_2Cl_2. ○ = Ar^F-Ar^F, □ = CH_3CN-$B(Ar^F)_3$, ● = $B(Ar^F)_4^-$, x = internal standard (1,4-ditrimethylsilylbenzene). The resonance of the methyl protons of CH_3CN-$B(Ar^F)_3$ appeared at δ = 2.78 at -50 °C, although the crude sample showed only a broadened methyl proton resonance at room temperature.

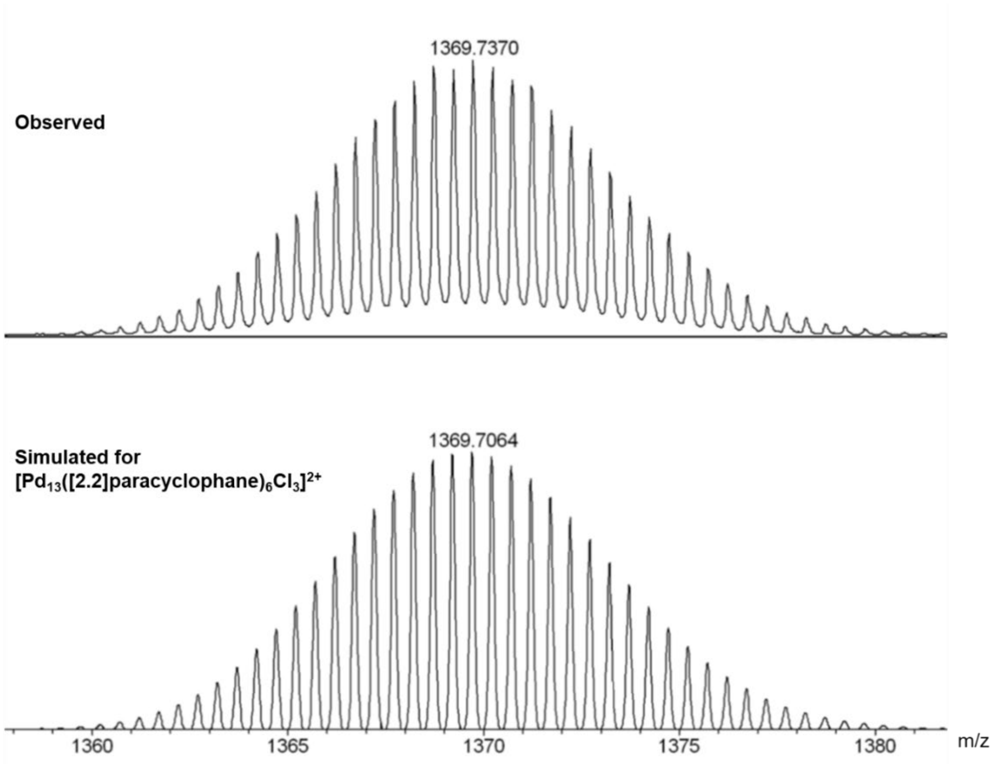

Figure S3. ESI-MS spectrum of $[Pd_{13}(\mu_4\text{-PCP})_6(\mu\text{-Cl})_3][B(Ar^F)_4]_2$ (**2**).

Synthesis of $[Pd_{17}(\mu_3\text{-PCP})_8(\mu_4\text{-Cl})_2][B(Ar^F)_4]_3$ (**3**)

The crude mixture of **2** and **3** (377 mg, **2**:**3** = 56:44) was obtained according to the procedure described for the preparation of **2** (2.00 g of $[Pd_3(\mu_3\text{-PCP})_2(CH_3CN)_3][B(Ar^F)_4]_2$ (**1**) (7.74 x 10^{-1} mmol) was used.). The mixture was dissolved in *ca.* 5 mL of 1,2-dichloroethane, and then the solution was precipitated with *ca.* 10 mL of Et_2O. The supernatant was evaporated, and the resultant solid was dissolved in 1,2-dichloroethane and the solution was precipitated with Et_2O to afford a black powder of complexes **2** and **3** (229 mg, complex **2**:**3** = 33:67). Recrystallization from 1,2-dichloroethane/Et_2O gave black crystals. At this point, the ^1H NMR spectrum of the crude product showed broad signals, presumably due to the contamination of an oxidized product of **3**. The crystals were dissolved in CH_2Cl_2 and stirred for 30 min at ambient temperature in H_2 atmosphere. The reaction mixture was filtered through Celite, and the filtrate was evaporated. The resultant solid was dissolved in 1,2-dichloroethane and the solution was reprecipitated with Et_2O, followed by washed with Et_2O to afford a black powder of complex **3** (67.2 mg, 1.10 x 10^{-2} mmol, 8%). ^1H NMR (400 MHz, CD_2Cl_2, 5.0 mM, 25 °C): δ 9.62 (br, 32H, cyclophane CH_2), 7.77 (s, 24H, *o*-BAr^F_4), 7.58 (s, 12H, *p*-BAr^F_4), 6.76 (s, 32H, cyclophane Ar*H*), 2.65 (br, 32H, cyclophane CH_2), -6.41 (br, 32H, cyclophane Ar*H*). $^{13}C\{^1H\}$ NMR (101 MHz, CD_2Cl_2, 25 °C): δ 168.5 (br, cyclophane *Ar*H), 162.1 (q, $^1J_{C\text{-}B}$ = 50 Hz, *ipso*-BAr^F), 141.3 (s, cyclophane *Ar*H), 139.2 (s, cyclophane cyclophane *ipso*-*Ar*), 135.2 (s, *o*-BAr^F), 129.2 (q, $^2J_{C\text{-}F}$ = 29 Hz, *m*-BAr^F), 125.0 (q, $^1J_{C\text{-}F}$ = 274 Hz, CF_3), 117.9 (s, *p*-BAr^F), 89.3 (s, cyclophane *C*H_2), 10.1 (s, cyclophane *C*H_2). Signals for one set of *ipso*-carbons in cyclophane ligands were not observed. Anal. Calcd. For. $C_{224}H_{164}B_3Cl_2F_{72}Pd_{17}$: C, 43.85; H, 2.69. Found: C, 43.87; H, 2.48.

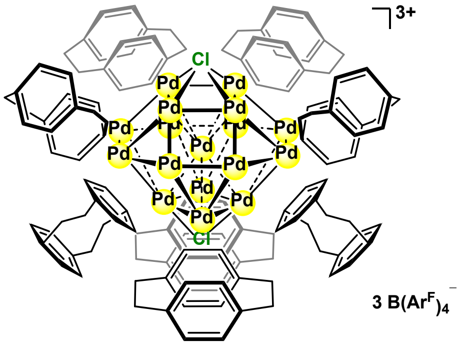

3

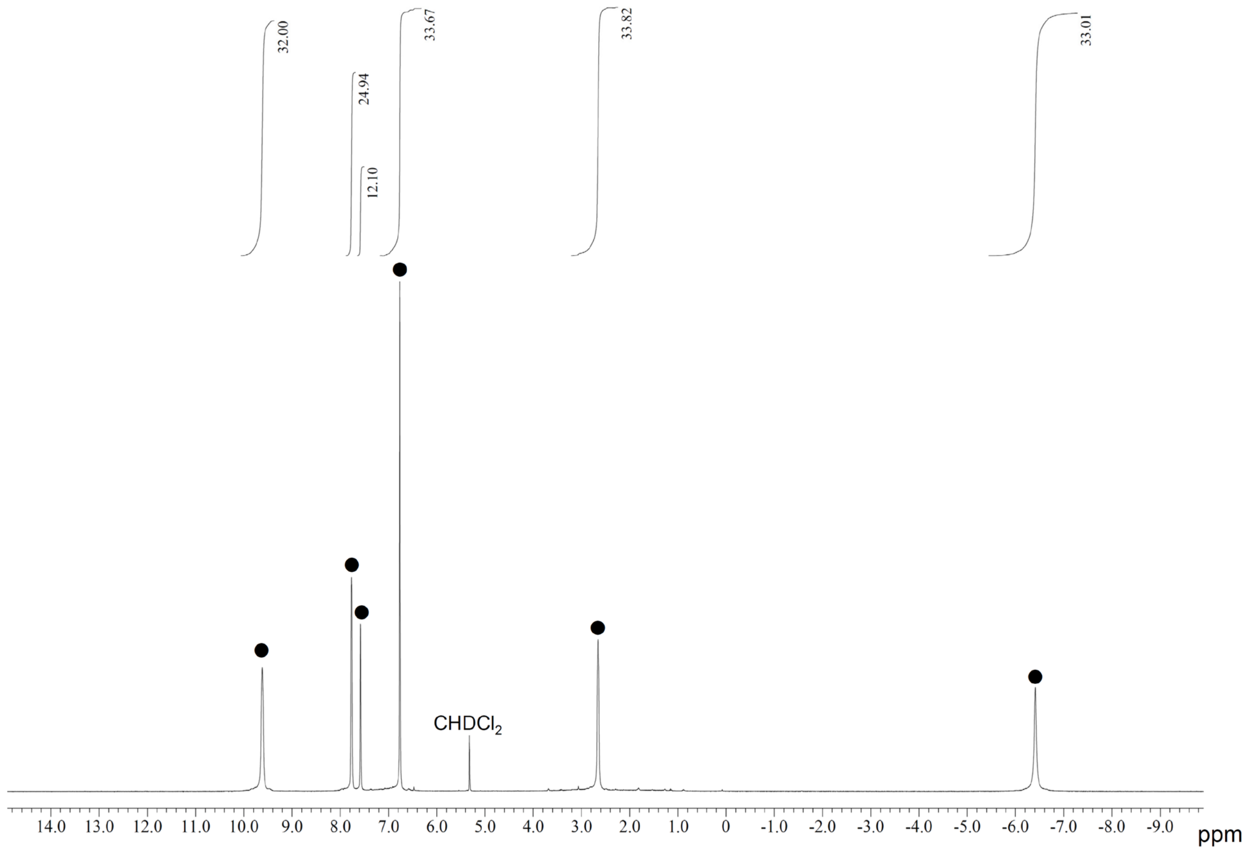

Figure S4. ^{1}H NMR spectrum of $[Pd_{17}(\mu_3\text{-PCP})_8(\mu_4\text{-Cl})_2][B(Ar^F)_4]_3$ (**3**). • = $[Pd_{17}(\mu_3\text{-PCP})_8(\mu_4\text{-Cl})_2][B(Ar^F)_4]_3$.

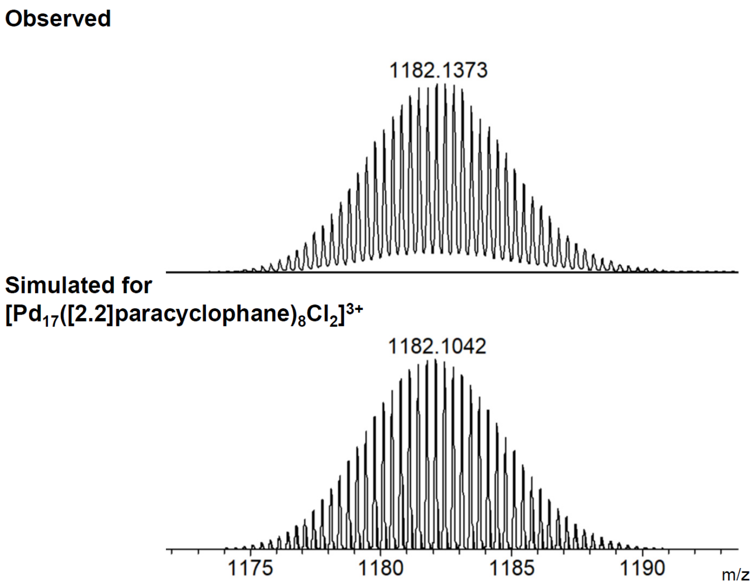

Figure S5. ESI-MS spectrum of $[Pd_{17}(\mu_3\text{-PCP})_8(\mu_4\text{-Cl})_2][B(Ar^F)_4]_3$ (**3**) (range: *m/z* 1170-1195).

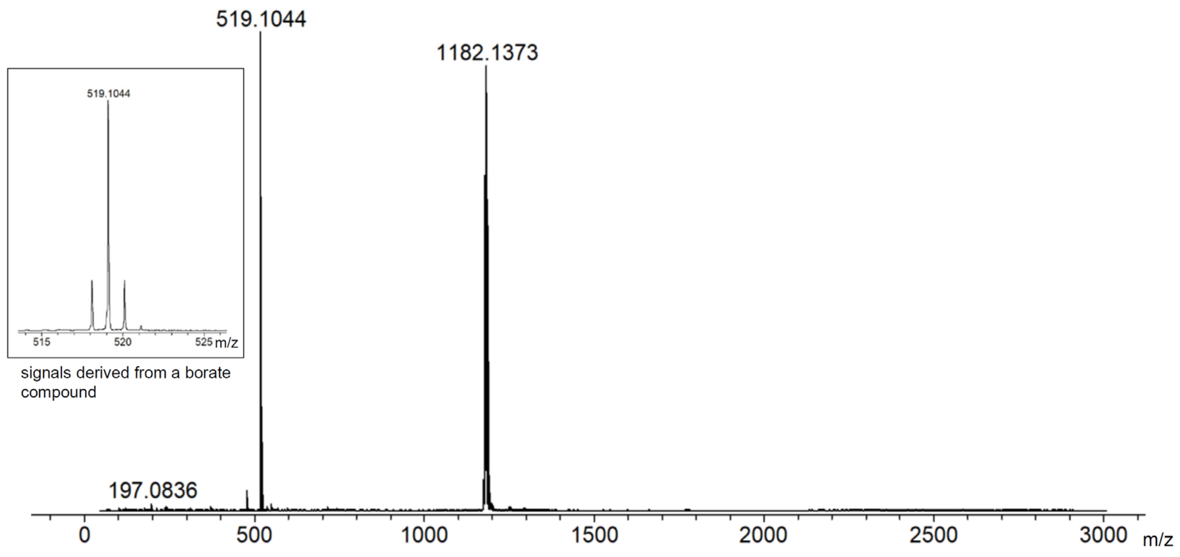

Figure S6. ESI-MS spectrum of $[Pd_{17}(\mu_3\text{-PCP})_8(\mu_4\text{-Cl})_2][B(Ar^F)_4]_3$ (**3**) (range: *m/z* 0-3000).

Synthesis of $[Pd_{13}(\mu_4\text{-PCP})_6(\mu\text{-Cl})_3][B(Ar^F)_4]$ (**2'**)

To a solution of $[Pd_{13}(\mu_4\text{-PCP})_6(\mu\text{-Cl})_3][B(Ar^F)_4]_2$ (**2**) (5.0 mg, 1.1 μmol) was added Et_3N (0.18 μL, 1.3 μmol) in CD_2Cl_2. The mixture was stood at ambient temperature for 10 min to give complex **2'** in 96% yield (the yield was determined by ^{1}H NMR). Complex **2'** was not isolated due to the formation of insoluble materials during washing the crude products; however, crystals of complex **2'** was obtained by recrystallization from 1,2-dichloroethane/Et_2O. ^{1}H NMR (400 MHz, CD_2Cl_2, 25 °C): δ 7.73 (s, 8H, *o*-$BAr^F{}_4$), 7.58 (s, 4H, *p*-$BAr^F{}_4$), 7.02 (s, 24H, cyclophane Ar*H*), 4.66 (s, 24H, cyclophane Ar*H*), 3.09 (m, 24H, cyclophane CH_2), 2.22 (m, 24H, cyclophane CH_2). $^{13}C\{^1H\}$ NMR (101 MHz, CD_2Cl_2, 25 °C) δ 162.1 (q, $^1J_{C\text{-}B}$ = 50 Hz, *ipso*-BAr^F), 139.4 (s, cyclophane *ipso*-*Ar*H), 135.2 (s, *o*-BAr^F), 132.0 (s, cyclophane *Ar*H), 129.2 (q, $^2J_{C\text{-}F}$ = 35 Hz, *m*-BAr^F), 125.0 (q, $^1J_{C\text{-}F}$ = 273 Hz, CF_3), 118.5 (s, cyclophane *ipso*-*Ar*), 117.8 (s, *p*-BAr^F), 88.2 (s, cyclophane *Ar*H), 37.9 (s, cyclophane *C*H_2), 35.3 (s, cyclophane *C*H_2).

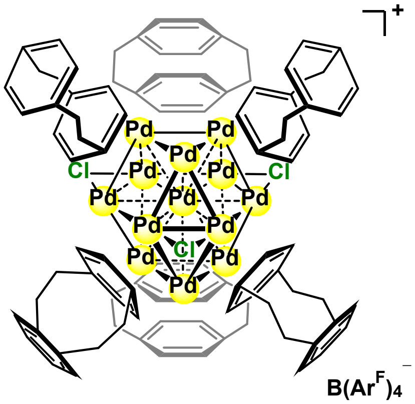

2'

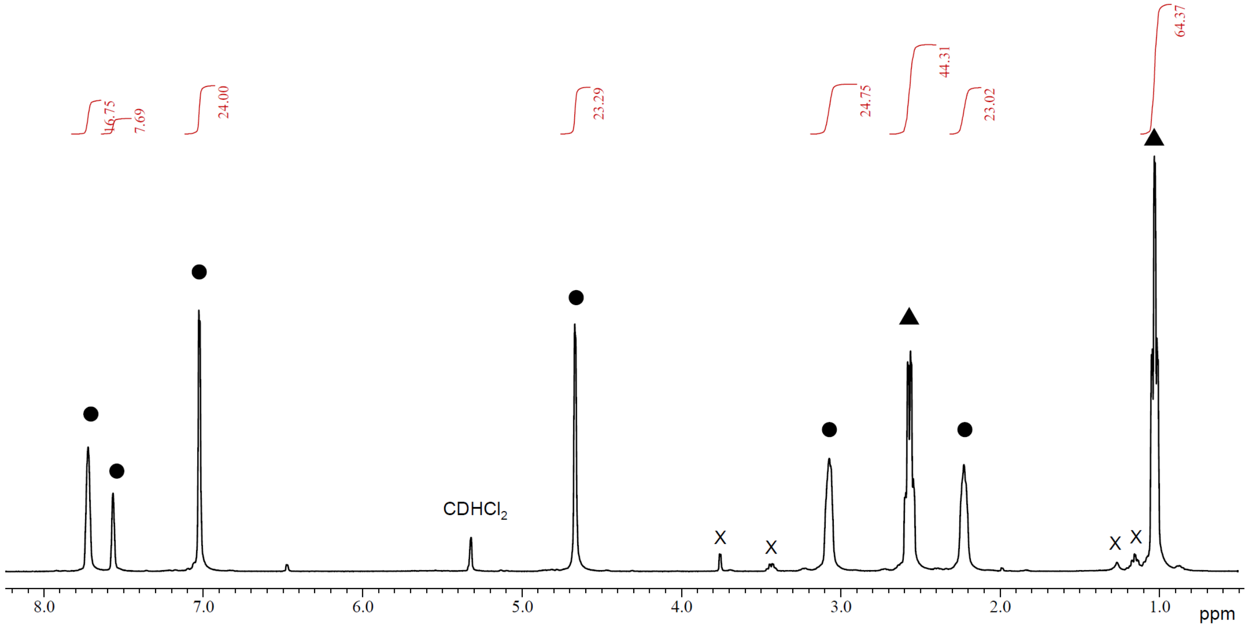

Figure S7. ^{1}H NMR spectrum of a mixture of $[Pd_{13}(\mu_4\text{-PCP})_6(\mu\text{-Cl})_3][B(Ar^F)_4]$ (**2'**). • = $[Pd_{13}(\mu_4\text{-PCP})_6(\mu\text{-Cl})_3][B(Ar^F)_4]$, ▲ = Products derived from Et_3N. x = impurities.

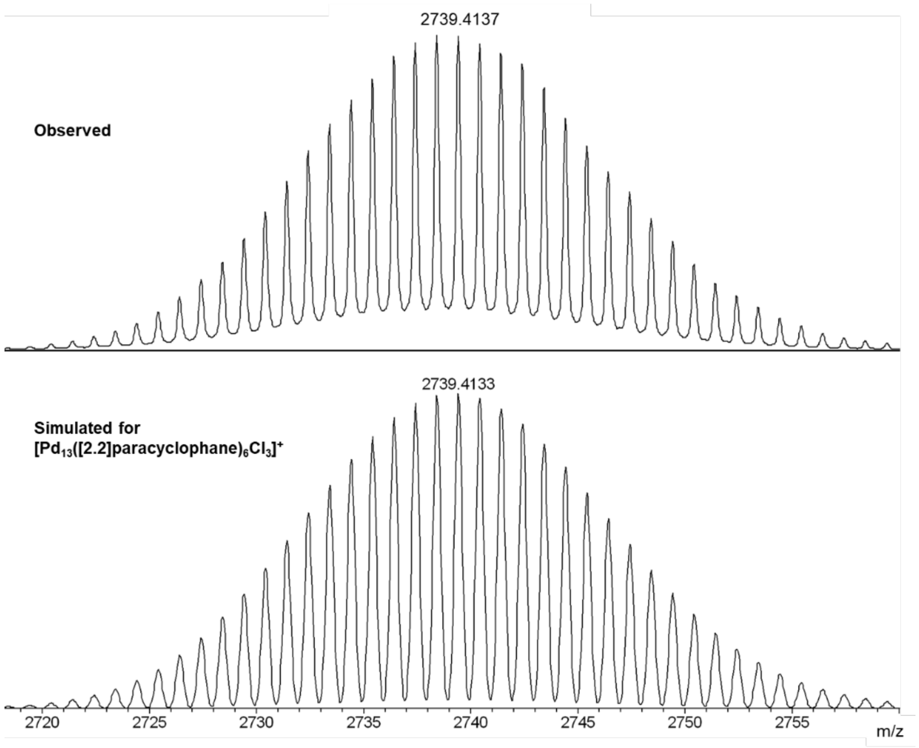

Figure S8. ESI-MS spectrum of complex [Pd_{13}(μ_4-PCP)$_6$(μ-Cl)$_3$][B(Ar^F)$_4$] (**2'**).

Synthesis of $[Pd_{17}(PCP)_8Cl_2][BAr^F{}_4]_4$ (**3'**)

To a solution of $[Pd_{17}(\mu_3\text{-}PCP)_8(\mu_4\text{-}Cl)_2][B(Ar^F)_4]_3$ (**3**) (30.0 mg, 4.89 x 10^{-3} mmol) in CH_2Cl_2 was added $AgPF_6$ (1.85 mg, 7.32 x 10^{-3} mmol, 1.5 eq) and $NaB(Ar^F)_4$ (6.50 mg, 7.33 x 10^{-3} mmol, 1.5 eq), and then the mixture was stirred for 10 min at ambient temperature. The reaction mixture was filtered through Celite. The filtrate was concentrated *in vacuo*, precipitated with Et_2O, and washed with Et_2O to afford a black powder. At this point, signals of starting material complex **3** was observed in ^1H NMR of the crude product, therefore the crude was reacted with an additional oxidant as follows. The powder was dissolved in dichloromethane, and the solution was added $AgPF_6$ (1.01 mg, 3.99 x 10^{-3} mmol, 0.8 eq). After stirring for 10 min at ambient temperature, the reaction mixture was filtered through Celite. The filtrate was concentrated in vacuo, precipitated with Et_2O, and washed with Et_2O to afford a black powder of complex **3'** (22.7 mg, 3.24 x 10^{-3} mmol, 66%). ^1H NMR (400 MHz, CD_2Cl_2, 25 °C): δ 7.78 (s, 32H, *o*-$BAr^F{}_4$), 7.59 (s, 16H, *p*-$BAr^F{}_4$), 6.84 (s, 32H, cyclophane Ar*H*), 4.35 (m, 32H, cyclophane C*H*$_2$), 3.16 (m, 32H, cyclophane C*H*$_2$), 2.62 (s, 32H, cyclophane Ar*H*). $^{13}C\{^1H\}$ NMR (101 MHz, CD_2Cl_2, 25 °C) δ 162.2 (q, $^1J_{C\text{-}B}$ = 50 Hz, *ipso*-BAr^F), 138.7 (s, cyclophane *ipso*-Ar), 135.2 (s, *o*-BAr^F), 134.6 (s, cyclophane *Ar*H), 129.2 (q, $^2J_{C\text{-}F}$ = 34 Hz, *m*-BAr^F), 125.1 (q, $^1J_{C\text{-}F}$ = 273 Hz, *C*F_3), 117.9 (s, *p*-BAr^F), 103.1 (s, cyclophane *Ar*H), 46.9 (s, cyclophane *C*H_2), 32.1 (s, cyclophane *C*H_2). Anal. Calcd. For. Signals for one set of *ipso*-carbons in cyclophane ligands were not observed. $C_{256}H_{176}B_4Cl_2F_{96}Pd_{17}$: C, 43.93; H, 2.53. Found: C, 43.86; H, 2.44.

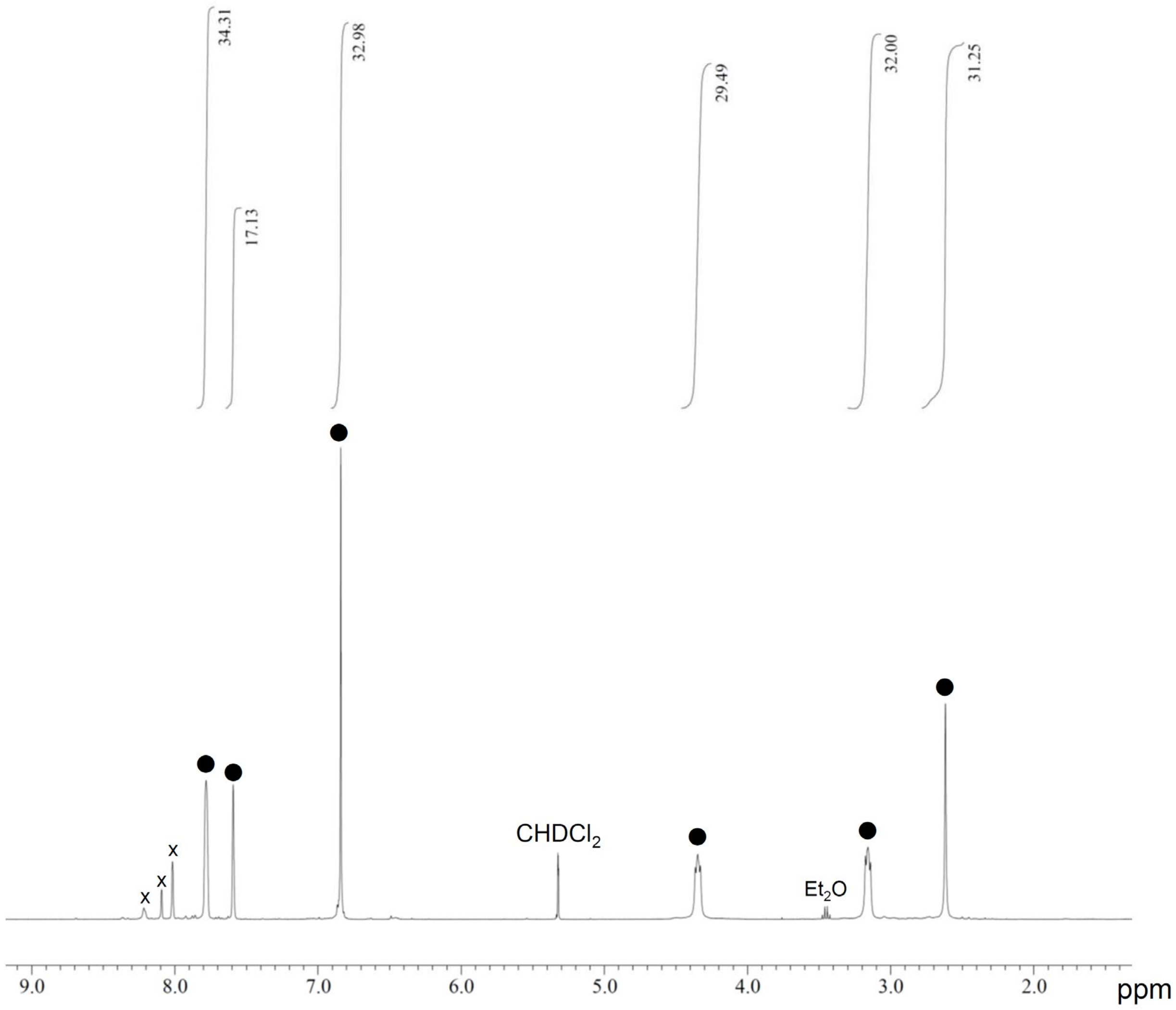

Figure S9. ^{1}H NMR spectrum of complex $[Pd_{17}(PCP)_8Cl_2][B(Ar^F)_4]_4$ (**3'**). • = $[Pd_{17}(PCP)_8Cl_2][B(Ar^F)_4]_4$, × = impurities derived from $B(Ar^F)_4^-$.

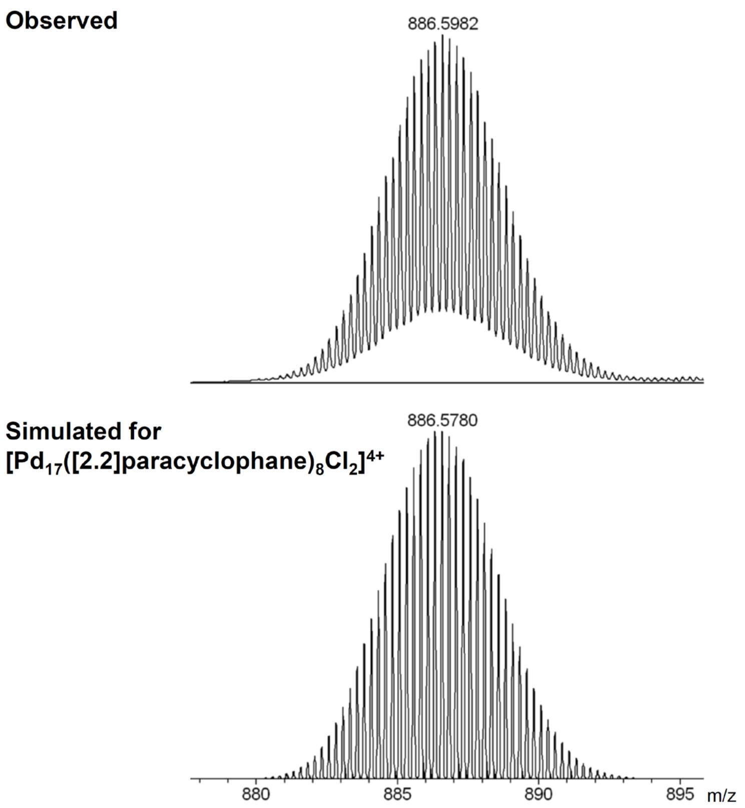

Figure S10. ESI-MS spectrum of complex $[Pd_{17}(PCP)_8Cl_2][B(Ar^F)_4]_4$ (**3'**).

Synthesis of $[Pd_3(\mu_3\text{-PCP})_2(PhCN)_3][PF_6]_2$ (**1'**)

To a solution of [2.2]paracyclophane (PCP) (1.00 g, 4.80 mmol) was added $[Pd_2(PhCN)_6][PF_6]_2$ (556 mg, 4.95 x 10^{-1} mmol) and $Pd_2(dba)_3{\cdot}CHCl_3$ (256 mg, 2.47 x 10^{-1} mmol), and then the mixture was stirred for overnight at ambient temperature. After evaporation of the reaction mixture, the resultant solid was dissolved in CH_3NO_2 and the solution was filtered. The filtrate was evaporated, the resultant solid was dissolved in CH_2Cl_2. To this solution was added Pd(2-norbornene)$_3$ (199 mg, 5.11 x 10^{-1} mmol), and then the mixture was stirred for 10 min at ambient temperature. The reaction mixture was filtered. The filtrate was precipitated with n-hexane to afford a brown powder of complex **1'** including $[Pd_2(\mu_3\text{-PCP})_2(PhCN)_3][PF_6]_2$ (Pd_3/Pd_2 =77/23), (574 mg, 3.41 x 10^{-1} mmol, 69%). ^{1}H NMR (400 MHz, CD_3NO_2, 25 °C): δ 7.90 (d, *J* = 7.6 Hz, 6H, *o-Ph*CN), 7.85 (t, *J* = 7.6 Hz, 3H, *p-Ph*CN), 7.67 (dd, *J* = 7,6 and 7.6 Hz, 6H, *m-Ph*CN), 6.98 (s, 8H, cyclophane Ar*H*), 5.81 (s, 8H, cyclophane Ar*H*), 3.18 (m, 8H, cyclophane C*H*$_2$), 2.75 (m, 8H, cyclophane C*H*$_2$). ^{13}C{^{1}H} NMR (101 MHz, CD_3NO_2, 25 °C) δ 140.9 (s, cyclophane *ipso*-Ar), 136.2 (s, *p-Ph*CN), 134.3 (s, *o-Ph*CN), 133.5 (s, cyclophane *Ar*H), 131.0 (s, *m-Ph*CN), 128.7 (s, Ph*C*N), 119.8 (s, cyclophane *ipso*-Ar), 111.5 (s, *ipso-Ph*CN), 93.0 (s, cyclophane *Ar*H), 37.0 (s, cyclophane *C*H$_2$), 36.0 (s, cyclophane *C*H$_2$).

Electrochemical Measurements

The cyclic voltammograms were obtained by ALS 600A electrochemical analyzer. The cell was placed in a grove box under an atmosphere of N_2 at 296 K. A 1.6 mm diameter Pt working electrode or 3 mm diameter glassy carbon electrode, Pt wire counter electrode, and Ag/Ag^+ reference electrode were used. The electrochemical measurement was made in CH_2Cl_2 with 0.5 mM of complex **2** or **3** containing [^{n}Bu$_4$N][BAr$^F{}_4$] (0.1 M). A scan rate is 100 mV s^{-1}.

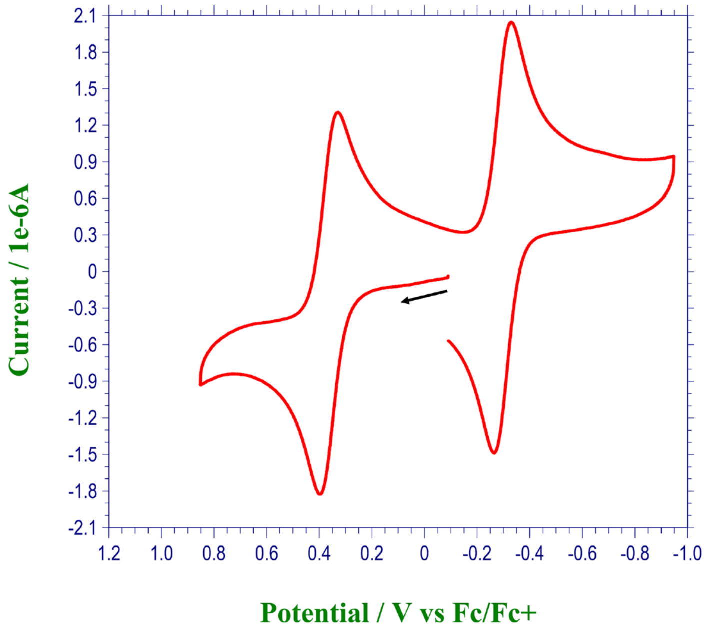

Figure S11. The cyclic voltammogram of $[Pd_{13}(\mu_4\text{-PCP})_6(\mu\text{-Cl})_3][B(Ar^F)_4]_2$ (**2**) in CH_2Cl_2. An arrow indicates sweep direction.

For **2**,

$E_{1/2}$ = +0.36 V, $[Pd_{13}(PCP)_6Cl_3]^{3+}/[Pd_{13}(PCP)_6Cl_3]^{2+}$;

$E_{1/2}$ = -0.30 V, $[Pd_{13}(PCP)_6Cl_3]^{2+}/[Pd_{13}(PCP)_6Cl_3]^{+}$

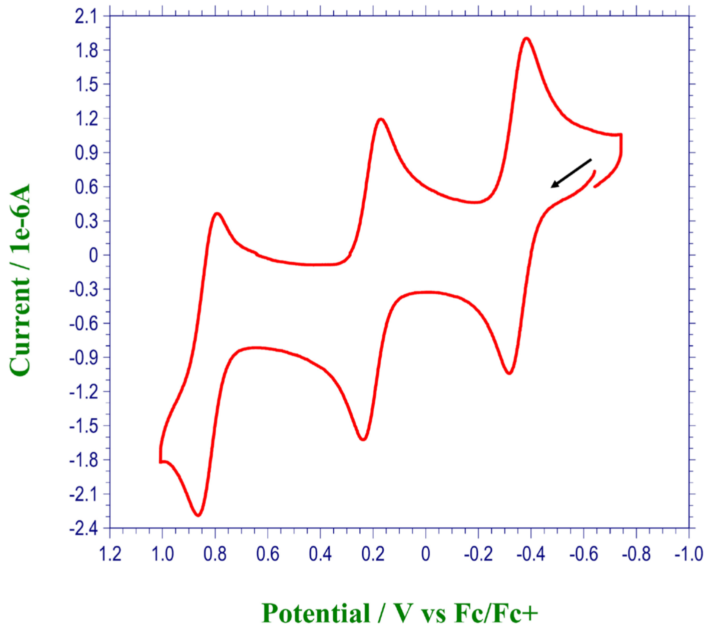

Figure S12. The cyclic voltammogram of $[Pd_{17}(\mu_3\text{-PCP})_8(\mu_4\text{-Cl})_2][B(Ar^F)_4]_3$ (**3**) in CH_2Cl_2. An arrow indicates sweep direction.

For **3**,

$E_{1/2}$ = +0.83 V, $[Pd_{17}(PCP)_8Cl_2]^{5+}/[Pd_{17}(PCP)_8Cl_2]^{4+}$;

$E_{1/2}$ = +0.20 V, $[Pd_{17}(PCP)_8Cl_2]^{4+}/[Pd_{17}(PCP)_8Cl_2]^{3+}$;

$E_{1/2}$ = -0.35 V, $[Pd_{17}(PCP)_8Cl_2]^{3+}/[Pd_{17}(PCP)_8Cl_2]^{2+}$

X-ray Crystallographic analyses

The measurements for **2**, **3**, and **2'** were performed on Rigaku XtaLAB Synergy with graphite-monochromated Cu-Kα (1.54184 Å) radiation. The structure was solved by direct methods (SHELXT)[4] and refined on F^2 by full-matrix least-squares methods; using SHELXL (2017/1 or 2018/3).[5] Non-hydrogen atoms were anisotropically refined. Hydrogen atoms were included in the refinement on calculated positions riding on their carrier atoms. Hydrogen atoms on coordinatied arene C–H moieties in cyclophane ligands were constrained with riding model instructions of AFIX13 or AFIX43 according to the peaks observed by difference Fourier synthesis maps. The function minimized was $[\Sigma w(F\mathrm{o}^2 - F\mathrm{c}^2)^2]$ ($w = 1 / [\sigma^2 (F\mathrm{o}^2) + (a\mathrm{P})^2 + b\mathrm{P}]$), where $\mathrm{P} = (\mathrm{Max}(F\mathrm{o}^2,0) + 2F\mathrm{c}^2) / 3$ with $\sigma^2(F\mathrm{o}^2)$ from counting statistics. The function $R1$ and $wR2$ were $(\Sigma||F\mathrm{o}| - |F\mathrm{c}||) / \Sigma|F\mathrm{o}|$ and $[\Sigma w(F\mathrm{o}^2 - F\mathrm{c}^2)^2 / \Sigma(wF\mathrm{o}^4)]^{1/2}$, respectively. The ORTEP-3 program was used to draw the molecules.[6] Crystal data for the structures reported in this paper have been deposited in the Cambridge Crystallographic Database Centre: CCDC 2171749, 2171750 and 2171751.

X-ray Crystallographic Data

Crystal data for **2**: $C_{160}H_{120}B_2Cl_3F_{48}Pd_{13}$, M_r = 4465.72, *monoclinic*, space group *C*2/*c* (no. 15). *a* = 36.2710(4) Å, *b* = 13.7483(2) Å, *c* = 33.9304(4) Å, β = 104.5650(10)°, Z = 4, *V* = 16376.1(4) Å^3, *F*(000) = 8684.0, D_c = 1.811 g cm^{-3}, μ(CuKα) = 12.574 mm^{-1}, *T* = 93.15 K, 113731 reflections collected, 15617 unique (R_{int} = 0.0747), 1019 variables refined with 13431 reflections with $I > 2\sigma(I)$ to *R* = 0.0587. CCDC 2171749.

Crystal data for **3**: $C_{224}H_{164}B_3Cl_2F_{72}Pd_{17}$, M_r = 6135.67, *monoclinic*, space group $P2_1/n$ (no. 14). *a* = 22.03694(8) Å, *b* = 25.97458(10) Å, *c* = 39.43602(14) Å, *β* = 104.4915(4)º, Z = 4, *V* = 21855.01(14) Å^3, *F*(000) = 11948, D_c = 1.865 g cm^{-3}, μ(CuKα) = 12.574 mm^{-1}, *T* = 93.15 K, 362170 reflections collected, 44833 unique (R_{int} = 0.0638), 2890 variables refined with 40199 reflections with $I > 2\sigma(I)$ to *R* = 0.0496. CCDC 2171750.

Crystal data for **2'**: $C_{134}H_{120}BCl_9F_{24}Pd_{13}$, M_r = 3899.35, *orthorhombic*, space group $Pna2_1$ (no. 33), *a* = 28.3118(3) Å, *b* = 13.75670(10) Å, *c* = 33.6236(3) Å, Z = 4, *V* = 13095.6(2) Å^3, *F*(000) = 7584.0, *D*c = 1.978 g cm^{-3}, μ(CuKα) = 16.429 mm^{-1}, *T* = 93.15 K, 44142 reflections collected, 18151 unique (R_{int} = 0.0617), 1221 variables refined with 16958 reflections with $I > 2\sigma(I)$ to *R* = 0.0584. CCDC 2171751.

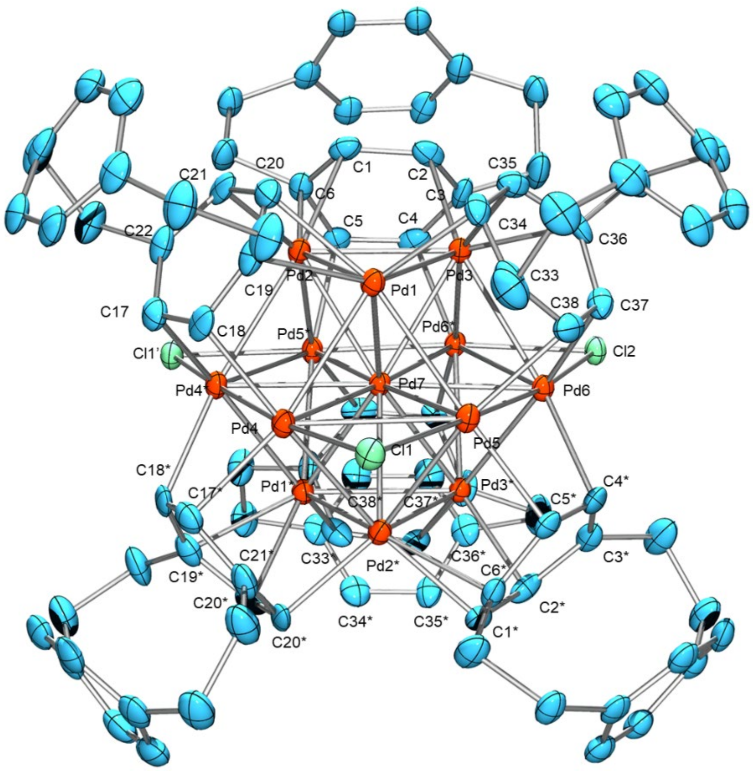

Figure S13. An ORTEP of complex **2** (30% probability ellipsoid, $B(Ar^F)_4$ anions and protons are omitted for clarity).

Table S1. Selected Bond Distances (Å) of Complex **2**.

Pd1–Pd2	2.7473(6)	Pd5–Cl1	2.4970(16)	Pd6–C37	2.181(7)
Pd2–Pd3	2.7523(6)	Pd6–Cl2	2.4980(16)	C1–C2	1.457(11)
Pd3–Pd1	2.7457(6)	Pd2–C1	2.229(6)	C2–C3	1.416(11)
Pd4–Pd5	3.0167(6)	Pd2–C6	2.328(6)	C3–C4	1.435(11)
Pd6–Pd6*	3.0146(8)	Pd3–C2	2.193(6)	C4–C5	1.402(10)
Pd4–Pd4*	2.5392(9)	Pd3–C3	2.579(7)	C5–C6	1.441(10)
Pd5–Pd6	2.5477(6)	Pd6–C4*	2.238(6)	C6–C1	1.394(11)
Pd1–Pd4	2.7420(6)	Pd5–C5*	2.198(6)	C17–C18	1.435(11)
Pd1–Pd5	2.7215(6)	Pd1–C19	2.317(7)	C18–C19	1.422(11)
Pd2–Pd4*	2.7135(6)	Pd1–C20	2.261(7)	C19–C20	1.403(11)
Pd2–Pd5*	2.7482(7)	Pd2–C21	2.189(6)	C20–C21	1.439(12)
Pd3–Pd6	2.7636(6)	Pd2–C22	2.586(7)	C21–C22	1.410(12)
Pd3–Pd6*	2.7105(6)	Pd4–C17*	2.239(7)	C22–C17	1.406(11)
Pd7–Pd1	2.6778(5)	Pd4–C18	2.188(7)	C33–C34	1.403(11)
Pd7–Pd2	2.6728(5)	Pd1–C33	2.616(8)	C34–C35	1.431(11)
Pd7–Pd3	2.6702(6)	Pd1–C34	2.196(7)	C35–C36	1.404(11)
Pd7–Pd4	2.7829(7)	Pd3–C35	2.247(7)	C36–C37	1.448(11)
Pd7–Pd5	2.7816(4)	Pd3–C36	2.343(7)	C37–C38	1.398(11)
Pd7–Pd6	2.7837(6)	Pd5–C33	2.618(8)	C38–C33	1.429(11)
Pd4–Cl1	2.4749(16)	Pd5–C38	2.246(7)		

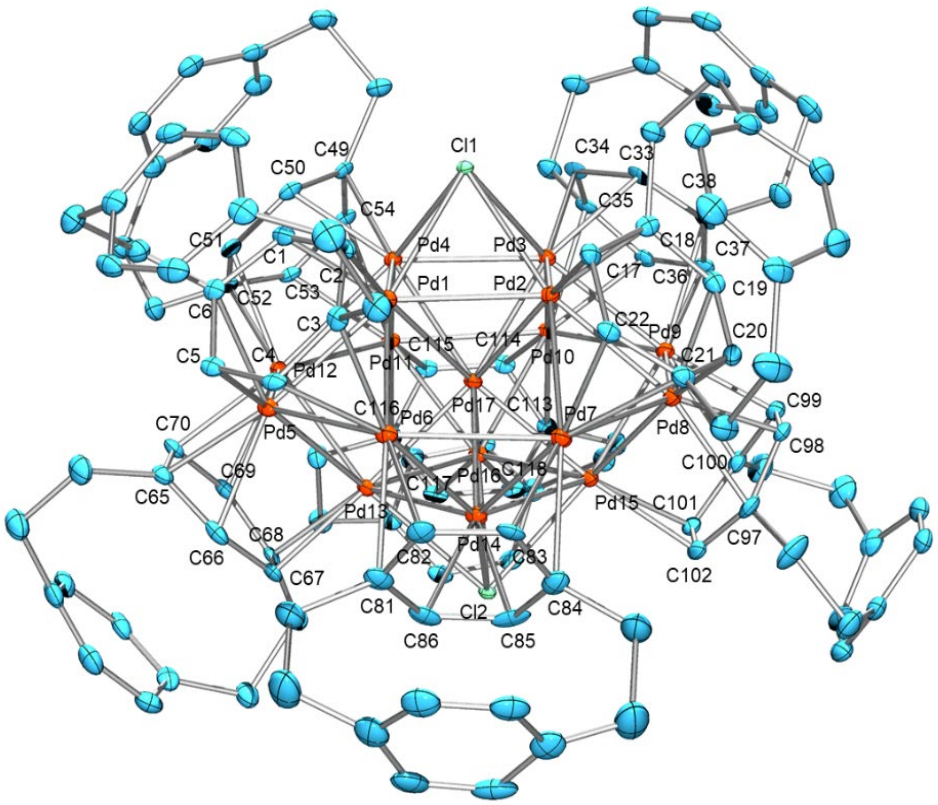

Figure S14. An ORTEP of complex **3** (30% probability ellipsoid, $B(Ar^F)_4$ anions and protons are omitted for clarity).

Table S2. Selected Bond Distances (Å) of Complex **3**.

Pd1–Pd2	2.7494(5)	Pd17–Pd5	3.7708(5)	Pd5–C65	2.293(5)	C37–C38	1.411(7)
Pd2–Pd3	2.7697(4)	Pd17–Pd6	3.6688(5)	Pd5–C66	2.222(5)	C38–C33	1.419(7)
Pd3–Pd4	2.6911(4)	Pd17–Pd7	3.7546(5)	Pd13–C67	2.171(4)	C49–C50	1.424(7)
Pd4–Pd1	2.6927(4)	Pd17–Pd8	3.6743(5)	Pd13–C68	2.207(4)	C50–C51	1.447(7)
Pd13–Pd14	2.7005(5)	Pd17–Pd9	3.6768(5)	Pd12–C69	2.257(4)	C51–C52	1.410(7)
Pd14–Pd15	2.7594(4)	Pd17–Pd10	3.6796(4)	Pd12–C70	2.291(4)	C52–C53	1.435(7)
Pd15–Pd16	2.7265(5)	Pd17–Pd11	3.5902(5)	Pd6–C81	2.293(5)	C53–C54	1.414(7)
Pd16-Pd13	2.7451(4)	Pd17–Pd12	3.6510(5)	Pd6–C82	2.219(5)	C54–C49	1.436(7)
Pd1–Pd5	2.6628(5)	Pd1–Cl1	2.6235(10)	Pd7–C83	2.246(5)	C65–C66	1.404(8)
Pd1–Pd6	2.6556(5)	Pd2–Cl1	2.6640(10)	Pd7–C84	2.295(5)	C66–C67	1.441(7)
Pd2–Pd7	2.7386(5)	Pd3–Cl1	2.6805(10)	Pd14–C85	2.174(5)	C67–C68	1.414(7)
Pd2–Pd8	2.6441(4)	Pd4–Cl1	2.6343(10)	Pd14–C86	2.229(5)	C68–C69	1.446(7)
Pd3–Pd9	2.6379(5)	Pd13–Cl2	2.7551(10)	Pd8–C97	2.266(5)	C69–C70	1.406(7)
Pd3–Pd10	2.6872(5)	Pd14–Cl2	2.6585(10)	Pd8–C98	2.230(5)	C70–C65	1.439(7)
Pd4–Pd11	2.6278(4)	Pd15–Cl2	2.6239(10)	Pd9–C99	2.199(5)	C81–C82	1.391(8)
Pd4–Pd12	2.7293(4)	Pd16–Cl2	2.6557(10)	Pd9–C100	2.305(5)	C82–C83	1.437(8)
Pd13–Pd5	2.7513(4)	Pd1–C1	2.191(5)	Pd15–C101	2.200(5)	C83–C84	1.410(8)
Pd13–Pd12	2.6369(5)	Pd1–C2	2.163(5)	Pd15–C102	2.216(5)	C84–C85	1.450(9)
Pd14–Pd6	2.6743(5)	Pd6–C3	2.294(5)	Pd10–C113	2.325(4)	C85–C86	1.426(9)
Pd14–Pd7	2.6516(5)	Pd6–C4	2.228(5)	Pd10–C114	2.231(5)	C86–C81	1.414(8)
Pd15–Pd8	2.6283(5)	Pd5–C5	2.212(4)	Pd11–C115	2.201(4)	C97–C98	1.418(7)
Pd15–Pd9	2.6718(4)	Pd5–C6	2.348(4)	Pd11–C116	2.279(4)	C98–C99	1.425(7)
Pd16–Pd10	2.6625(4)	Pd2–C17	2.204(5)	Pd16–C117	2.183(5)	C99–C100	1.401(8)
Pd16–Pd11	2.6542(5)	Pd2–C18	2.206(5)	Pd16–C118	2.190(4)	C100–C101	1.445(7)
Pd5–Pd6	2.8081(5)	Pd8–C19	2.238(5)	C1–C2	1.424(8)	C101–C102	1.429(8)
Pd6–Pd7	2.8694(5)	Pd8–C20	2.260(5)	C2–C3	1.429(7)	C102–C97	1.425(8)
Pd7–Pd8	2.7758(5)	Pd7–C21	2.457(5)	C3–C4	1.418(7)	C113–C114	1.411(7)
Pd8–Pd9	2.8937(5)	Pd7–C22	2.205(4)	C4–C5	1.452(8)	C114–C115	1.444(7)
Pd9–Pd10	2.8528(5)	Pd3–C33	2.172(4)	C5–C6	1.425(7)	C115–C116	1.421(7)
Pd10–Pd11	2.8568(5)	Pd3–C34	2.182(4)	C6–C1	1.431(7)	C116–C117	1.440(7)
Pd11–Pd12	2.7526(4)	Pd10–C35	2.288(4)	C17–C18	1.420(7)	C117–C118	1.429(7)
Pd12–Pd5	2.7526(5)	Pd10–C36	2.248(4)	C18–C19	1.439(7)	C118–C113	1.435(6)
Pd17–Pd1	2.5649(4)	Pd9–C37	2.223(4)	C19–C20	1.408(7)		
Pd17–Pd2	2.5992(4)	Pd9–C38	2.324(4)	C20–C21	1.433(7)		
Pd17–Pd3	2.5678(4)	Pd4–C49	2.237(4)	C21–C22	1.390(7)		
Pd17–Pd4	2.6136(4)	Pd4–C50	2.173(5)	C22–C17	1.464(7)		
Pd17–Pd13	2.5772(4)	Pd12–C51	2.209(4)	C33–C34	1.518(7)		
Pd17–Pd14	2.6007(4)	Pd12–C52	2.323(5)	C34–C35	1.429(7)		
Pd17–Pd15	2.5989(4)	Pd11–C53	2.246(5)	C35–C36	1.401(7)		
Pd17–Pd16	2.5670(4)	Pd11–C54	2.280(4)	C36–C37	1.447(8)		

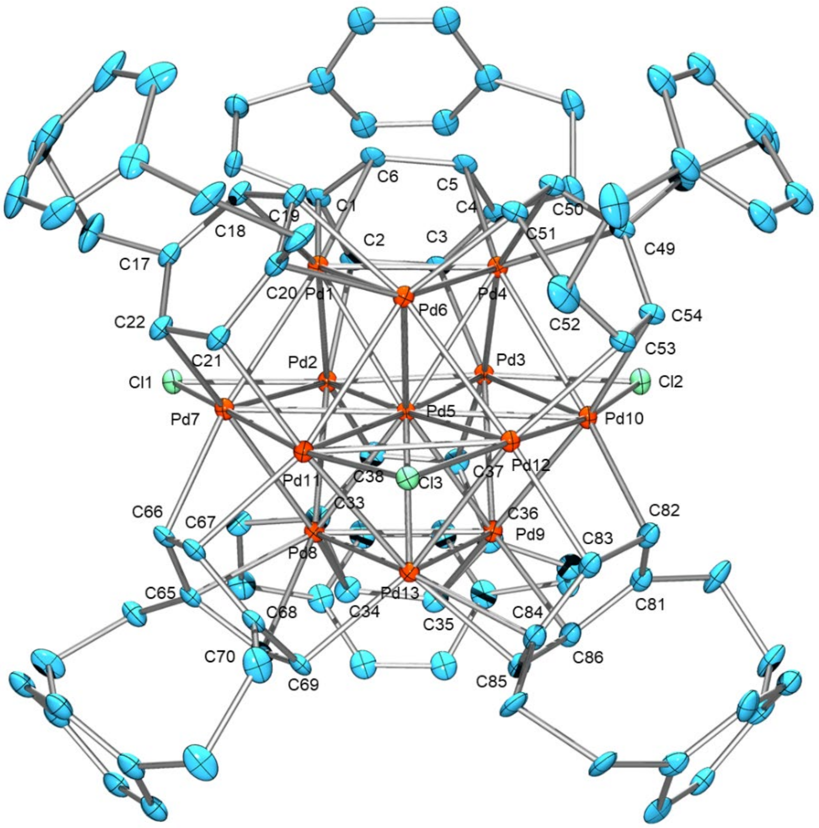

Figure S15. An ORTEP of complex **2'** (30% probability ellipsoid, $B(Ar^F)_4$ anions, solvent molecules, and protons are omitted for clarity).

Table S3. Selected Bond Distances (Å) of Complex **2'**.

Pd1–Pd6	2.8113(16)	Pd3–Cl2	2.492(4)	Pd9–C86	2.156(17)
Pd6–Pd4	2.7951(16)	Pd10–Cl2	2.502(4)	C1–C2	1.43(3)
Pd4–Pd1	2.8012(16)	Pd11–Cl3	2.538(4)	C2–C3	1.46(2)
Pd2–Pd7	3.0636(14)	Pd12–Cl3	2.525(4)	C3–C4	1.38(2)
Pd7–Pd11	2.5495(16)	Pd1–C1	2.298(16)	C4–C5	1.42(3)
Pd11–Pd12	3.0314(16)	Pd2–C2	2.189(16)	C5–C6	1.48(2)
Pd12–Pd10	2.5423(15)	Pd3–C3	2.249(16)	C6–C1	1.44(2)
Pd10–Pd3	3.0427(16)	Pd4–C4	2.637(17)	C17–C18	1.45(2)
Pd3–Pd2	2.5493(16)	Pd4–C5	2.189(16)	C18–C19	1.44(2)
Pd8–Pd13	2.7985(16)	Pd1–C6	2.212(16)	C19–C20	1.41(2)
Pd13–Pd9	2.8206(16)	Pd7–C17	2.617(17)	C20–C21	1.48(2)
Pd9–Pd8	2.8121(16)	Pd1–C18	2.145(17)	C21–C22	1.42(2)
Pd1–Pd2	2.7506(18)	Pd6–C19	2.260(17)	C22–C17	1.42(2)
Pd1–Pd7	2.6791(16)	Pd6–C20	2.254(17)	C33–C34	1.46(2)
Pd6–Pd11	2.7470(16)	Pd11–C21	2.221(17)	C34–C35	1.49(2)
Pd6–Pd12	2.6976(17)	Pd7–C22	2.234(17)	C35–C36	1.42(2)
Pd4–Pd10	2.7370(17)	Pd2–C33	2.627(17)	C36–C37	1.47(2)
Pd4–Pd3	2.6995(18)	Pd8–C34	2.165(16)	C37–C38	1.44(2)
Pd8–Pd2	2.6877(16)	Pd9–C35	2.240(17)	C38–C33	1.40(3)
Pd8–Pd7	2.7545(17)	Pd9–C36	2.298(16)	C49–C50	1.40(2)
Pd13–Pd11	2.6978(17)	Pd3–C37	2.204(17)	C50–C51	1.47(2)
Pd13–Pd12	2.7429(17)	Pd2–C38	2.222(16)	C51–C52	1.47(2)
Pd9–Pd10	2.6944(17)	Pd4–C49	2.319(16)	C52–C53	1.45(2)
Pd9–Pd3	2.7452(16)	Pd4–C50	2.254(16)	C53–C54	1.44(2)
Pd5–Pd1	2.6710(17)	Pd6–C51	2.185(16)	C54–C49	1.45(2)
Pd5–Pd2	2.8009(16)	Pd6–C52	2.647(16)	C65–C66	1.36(3)
Pd5–Pd3	2.8029(17)	Pd12–C53	2.237(16)	C66–C67	1.52(2)
Pd5–Pd4	2.6735(16)	Pd10–C54	2.205(16)	C67–C68	1.40(3)
Pd5–Pd6	2.6850(18)	Pd8–C65	2.287(17)	C68–C69	1.42(3)
Pd5–Pd7	2.7923(16)	Pd7–C66	2.192(17)	C69–C70	1.40(2)
Pd5–Pd8	2.6768(16)	Pd11–C67	2.218(17)	C70–C65	1.46(3)
Pd5–Pd9	2.6829(18)	Pd13–C69	2.190(17)	C81–C82	1.44(3)
Pd5–Pd10	2.7845(16)	Pd8–C70	2.253(17)	C82–C83	1.41(3)
Pd5–Pd11	2.8107(16)	Pd9–C81	2.606(16)	C83–C84	1.46(2)
Pd5–Pd12	2.7948(16)	Pd10–C82	2.215(17)	C84–C85	1.44(3)
Pd5–Pd13	2.6779(17)	Pd12–C83	2.168(17)	C85–C86	1.44(2)
Pd2–Cl1	2.498(4)	Pd13–C84	2.329(16)	C86–C81	1.46(2)
Pd7–Cl1	2.511(4)	Pd13–C85	2.263(17)		

Table S4. Summary of Pd–Pd bond distances, and bond angles of $[Pd_{13}(\mu_4\text{-PCP})_6(\mu\text{-}Cl)_3][B(Ar^F)_4]_2$ (**2**) and $[Pd_{13}(\mu_4\text{-PCP})_6(\mu\text{-}Cl)_3][B(Ar^F)_4]$ (**2'**).

bond distances		**2** (Å)	**2'** (Å)
Pd_{core}–$Pd_{top/bot}$[a]	max<br>min<br>average	2.6778(5)<br>2.6702(6)<br>2.674	2.6850(18)<br>2.6735(16)<br>2.678
Pd_{core}–Pd_{eq}	max<br>min<br>average	2.7837(6)<br>2.7816(4)<br>2.783	2.8107(16)<br>2.7845(16)<br>2.798
$Pd_{top/bot}$–Pd_{eq}	max<br>min<br>average	2.7636(6)<br>2.7105(6)<br>2.733	2.7545(17)<br>2.6791(16)<br>2.719
Pd_{eq}–Pd_{eq}[b]	max<br>min<br>average	3.0167(6)<br>3.0146(8)<br>3.016	3.0636(14)<br>3.0314(16)<br>3.046
Pd_{eq}–Pd_{eq}[c]	max<br>min<br>average	2.5477(6)<br>2.5392(9)<br>2.545	2.5495(16)<br>2.5423(15)<br>2.547
$Pd_{top/bot}$–$Pd_{top/bot}$	max<br>min<br>average	2.7523(6)<br>2.7473(6)<br>2.748	2.8206(16)<br>2.7951(16)<br>2.806
bond angles		**2** (°)	**2'** (°)
$Pd_{top/bot}$–Pd_{core}–$Pd_{top/bot}$[d]	max<br>min<br>average	63.49(5)<br>62.88(4)<br>63.2	62.012(13)<br>61.781(13)<br>61.86
$Pd_{top/bot}$–Pd_{core}–Pd_{eq}[d]	max<br>min<br>average	60.44(4)<br>58.68(4)<br>59.5	60.85(16)<br>59.611(16)<br>60.1
Pd_{eq}–Pd_{core}–Pd_{eq}[e]	max<br>min<br>average	66.42(4)<br>65.47(4)<br>66.0	65.657(15)<br>65.57(2)<br>65.6
Pd_{eq}–Pd_{core}–Pd_{eq}[f]	max<br>min<br>average	54.21(4)<br>54.12(4)<br>54.2	54.489(13)<br>54.29(2)<br>54.4

[a] Pd_{core}, $Pd_{top/bot}$, and Pd_{eq} mean the interstitial Pd, Pd atom belonging top or bottom three-member Pd ring, and the equatorial Pd atom (see Fig. S16 for Pd_{core}, $Pd_{top/bot}$, and Pd_{eq}). [b] Pd–Pd bond with Cl-bridge. [c] Pd–Pd bond without Cl-bridge. [d] Pd vertices on the same edge. [e] Pd vertices on the same edge with Cl-bridge. [f] Pd vertices on the same edge without Cl-bridge.

Computational Details

DFT calculations were carried out, using the B3PW91 functional[7-11] with the Grimme's empirical dispersion correction (D3)[12,13] and the Becke-Johnson (BJ) damping correction.[14-16] Geometry optimizations were performed in gas phase using LANL2DZ basis set for valence electrons of Pd atom with corresponding effective core potentials (ECPs)[17] for its core electrons and the 6-31G(d) basis sets for other atoms (H, C, and Cl). The optimization was performed in gas phase. The optimized geometries of $[Pd_{13}(\mu_4\text{-PCP})_6(\mu\text{-Cl})_3]^{2+}$ (**2^{2+}**), one-electron reduced $[Pd_{13}(\mu_4\text{-PCP})_6(\mu\text{-Cl})_3]^{+}$ (**2^{+}**), $[Pd_{17}(\mu_3\text{-PCP})_8(\mu_4\text{-Cl})_2]^{3+}$ (**3^{3+}**), and one-electron oxidized $[Pd_{17}(\mu_3\text{-PCP})_8(\mu_4\text{-Cl})_2]^{4+}$ (**3^{4+}**) agree with the experimental ones, as shown in Figure S14 and Table S4. Atomic charges were evaluated using Stuttgart-Dresden-Bonn (SDB) basis set[18] for Pd with corresponding ECPs and 6-311G(d) for other atoms. We compared NBO charges[19] and Hirshfeld charges[20] and used Hirshfeld charges for discussion here because the NBO charge of the core Pd atom (named Pd_{core}) of **2^{+}** seems overly negative; as shown in Table S5, the -0.489 *e* of the Pd_{core} NBO atomic charge of **2^{+}** seems too negative unreasonably, considering that the $Pd_{13}Cl_3$ core has plus one charge in a formal sense. These MO analysis and atomic charge analysis were carried out using the solvation model based on density (SMD) method to consider solvation effects by dichloromethane.[21]

The energy change of the reaction between three Cl radicals and $[Pd_{13}(PCP)_6]^{2+}$ (eq. 1) was evaluated using the solvation model based on density (SMD) method to consider solvation effects by dichloromethane because $[Pd_{13}(\mu\text{-Cl})_3(\mu_4\text{-PCP})_6]^{2+}$ is synthesized in dichloromethane solvent.

$$[Pd_{13}(PCP)_6]^{2+} + 3\ {\cdot}Cl \rightarrow [Pd_{13}(\mu\text{-Cl})_3(\mu_4\text{-PCP})_6]^{2+} \quad (1)$$

In this work, we used Gaussian16 program.[22]

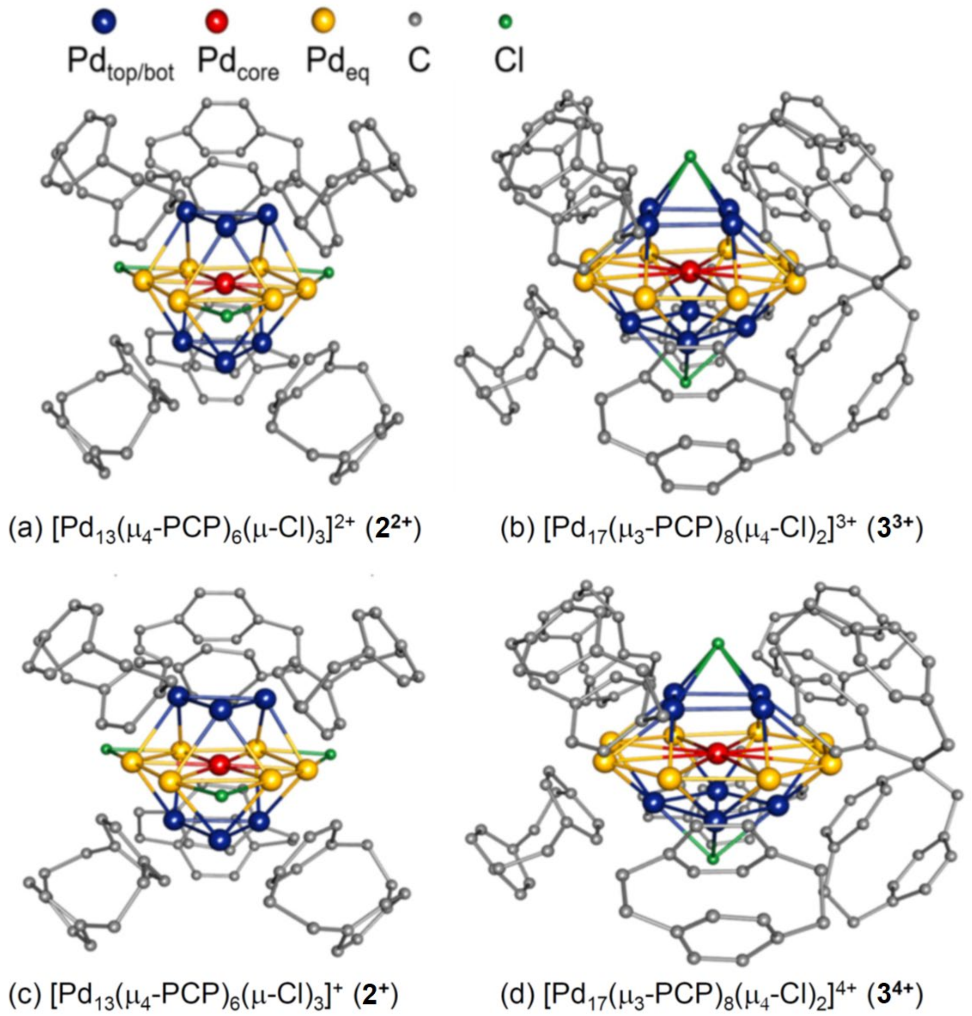

Figure S16. Optimized geometries of $[Pd_{13}(\mu_4\text{-PCP})_6(\mu\text{-Cl})_3]^{2+}$ ($\mathbf{2}^{2+}$), one-electron reduced $[Pd_{13}(\mu_4\text{-PCP})_6(\mu\text{-Cl})_3]^{+}$ ($\mathbf{2}^{+}$), $[Pd_{17}(\mu_3\text{-PCP})_8(\mu_4\text{-Cl})_2]^{3+}$ ($\mathbf{3}^{3+}$), and one-electron oxidized $[Pd_{17}(\mu_3\text{-PCP})_8(\mu_4\text{-Cl})_2]^{4+}$ ($\mathbf{3}^{4+}$). Important bond distances are presented in Table S4.

Table S5. Optimized Pd–Pd bond distances of $[Pd_{13}(\mu_4\text{-PCP})_6(\mu\text{-Cl})_3]^{2+}$ (**2**$^{2+}$), one-electron reduced $[Pd_{13}(\mu_4\text{-PCP})_6(\mu\text{-Cl})_3]^{+}$ (**2**$^{+}$), $[Pd_{17}(\mu_3\text{-PCP})_8(\mu_4\text{-Cl})_2]^{3+}$ (**3**$^{3+}$), and one-electron oxidized $[Pd_{17}(\mu_3\text{-PCP})_8(\mu_4\text{-Cl})_2]^{4+}$ (**3**$^{4+}$) in comparison with their experimental values.[a]

**Bonds**	**2**$^{2+}$ **Distance (Å)**		**3**$^{3+}$ **Distance (Å)**	
	**Expt.**[a]	**Calcd.**[a]	**Expt.**[a]	**Calcd.**[a]
**Pd$_{core}$–Pd$_{top/bot}$** [b]	2.674	2.693	2.586	2.604
**Pd$_{core}$–Pd$_{eq}$**	2.783	2.784	3.683	3.729
**Pd$_{top/bot}$–Pd$_{eq}$**	2.733	2.754	2.670	2.698
**Pd$_{eq}$–Pd$_{eq}$**[c]	3.016	3.013	–	–
**Pd$_{eq}$–Pd$_{eq}$** [d]	2.545	2.552	2.820	2.856
**Pd$_{top/bot}$–Pd$_{top/bot}$**	2.748	2.743	2.729	2.754
**Bonds**	**2**$^{+}$ **Distance (Å)**		**3**$^{4+}$ **Distance (Å)**	
	**Expt.**[a]	**Calcd.**[a]	**Expt.**	**Calcd.**[a]
**Pd$_{core}$–Pd$_{top/bot}$**	2.678	2.697	–	2.598
**Pd$_{core}$–Pd$_{eq}$**	2.798	2.800	–	3.685
**Pd$_{top/bot}$–Pd$_{eq}$**	2.719	2.738	–	2.684
**Pd$_{eq}$–Pd$_{eq}$**[c]	3.046	3.048	–	–
**Pd$_{eq}$–Pd$_{eq}$** [d]	2.547	2.549	–	2.822
**Pd$_{top/bot}$–Pd$_{top/bot}$**	2.806	2.810	–	2.727

[a] Experimental structure obtained by X-ray measurement. These are averaged values in each Pd–Pd bond type. [b] Pd$_{core}$, Pd$_{top/bot}$, and Pd$_{eq}$ mean the interstitial Pd, Pd atom belonging top or bottom three-member Pd ring, and the equatorial Pd atom (see Fig. S16 for Pd$_{core}$, Pd$_{top/bot}$, and Pd$_{eq}$). [c] Pd–Pd bond with Cl-bridge. [d] Pd–Pd bond without Cl-bridge.

Table S6. Hirshfeld and NBO charges of Pd_{core}, Pd_{eq}, $Pd_{top/bot}$, and $(C_{16}H_{16})_n$ (n = 6 or 8 for **2**$^+$ and **3**$^{4+}$, respectively) in $[Pd_{13}(\mu_4\text{-PCP})_6(\mu\text{-Cl})_3]^+$ (**2**$^+$) and $[Pd_{17}(\mu_3\text{-PCP})_8(\mu_4\text{-Cl})_2]^{4+}$ (**3**$^{4+}$).[a]

	**Hirshfeld charge**		**NBO charge**	
	**2**$^+$	**3**$^{4+}$	**2**$^+$	**3**$^{4+}$
**Pd_{core}** [b]	–0.089	–0.060	–0.489	–0.602
**Pd_{eq}** [b]	0.120	0.142	0.104	0.235
**$Pd_{top/bot}$** [b]	0.107	0.082	0.152	0.093
**$(C_{16}H_{16})_n$**	0.612	2.693	1.146	2.806

[a] The B3PW91-D3 functional was used with the SDD basis set for Pd and 6-311G(d) basis sets for other atoms. [b] Pd_{core}, $Pd_{top/bot}$, and Pd_{eq} mean the interstitial Pd atom, Pd atoms belonging top or bottom three-member Pd ring, and the equatorial Pd atoms (see Fig. S16 for Pd_{core}, $Pd_{top/bot}$, and Pd_{eq}). These are averaged values for equatorial Pd_6 or Pd_8 and top/bottom Pd_3 or Pd_4 members rings.

Table S7. The Hirshfeld charges of Pd_{13}, Pd_{17}, Cl_n (n= 3 or 2), and PCP ligands in $[Pd_{13}(\mu_4\text{-PCP})_6(\mu\text{-Cl})_3]^{2+}$ (**2**$^{2+}$) and $[Pd_{17}(\mu_3\text{-PCP})_8(\mu_4\text{-Cl})_2]^{3+}$ (**3**$^{3+}$).

$[Pd_{13}(\mu_4\text{-PCP})_6(\mu\text{-Cl})_3]^{2+}$ (**2**$^{2+}$)		$[Pd_{17}(\mu_3\text{-PCP})_8(\mu_4\text{-Cl})_2]^{3+}$ (**3**$^{3+}$)	
Pd_{13}	1.507	Pd_{17}	1.579
Cl_3	–0.826	Cl_2	–0.468
$C_{16}H_{16}$ (averaged)	0.220	$C_{16}H_{16}$ (averaged)	0.236

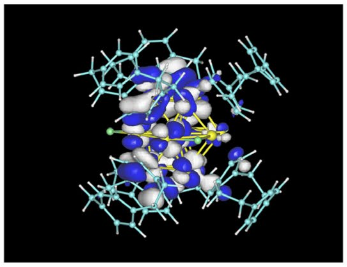

The 481st MO (-2.89 eV)

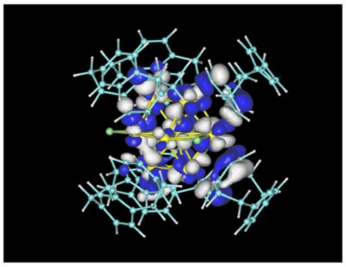

The 480th MO (-2.89 eV)

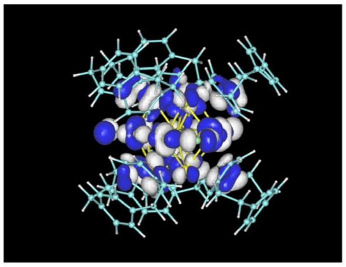

The 479th MO (-3.05 eV)
LUMO

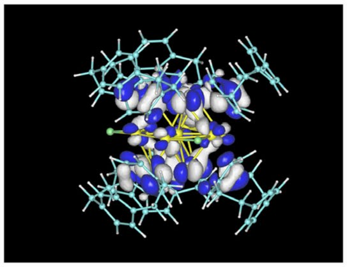

The 478th MO (-4.29 eV)
HOMO

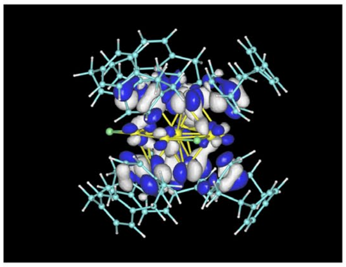

The 477th MO (-4.52 eV)

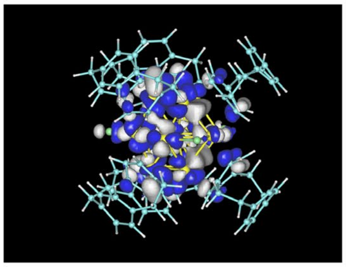

The 476th MO (-4.52 eV)

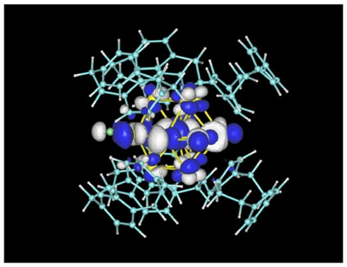

The 475th MO (-4.72 eV)

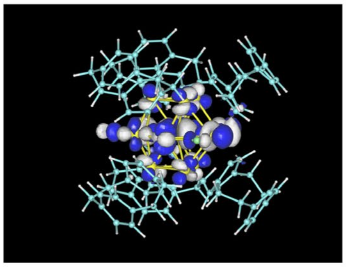

The 474th MO (-4.72 eV)

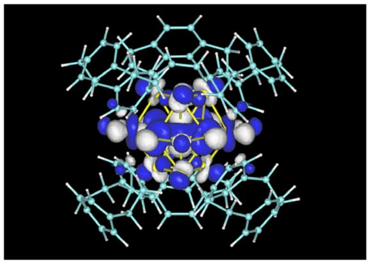

The 473rd MO (-4.90 eV)

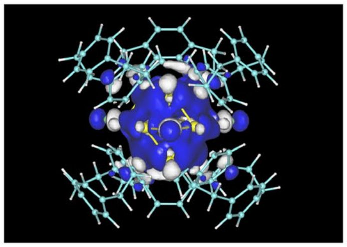

The 470th MO (-4.97 eV)

Figure S17. The Kohn-Sham orbitals of $[Pd_{13}(\mu_4\text{-PCP})_6(\mu\text{-Cl})_3]^+$ ($\mathbf{2}^+$).[a]

[a] Calculations were performed with the B3PW91-D3 functional using the LANL2DZ basis sets for Pd and 6-31G(d) basis sets for other atoms because clearer features of MO are generally shown by smaller basis sets than by larger basis sets with diffuse primitive functions. Numbers below figure represent the Kohn-Sham orbital energy (in eV unit).

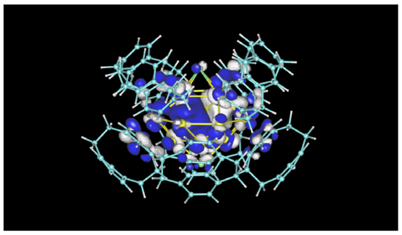

The 622nd MO (-3.63 eV)

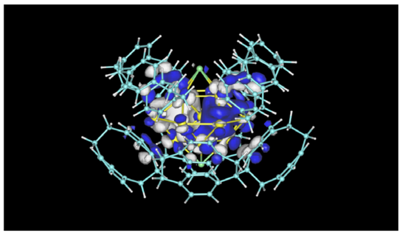

The 621st MO (-3.69 eV)

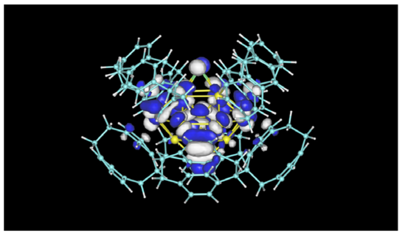

The 620th MO (-3.77 eV)

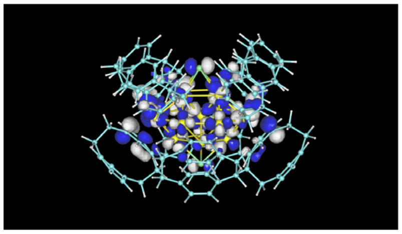

The 619th MO (-4.00 eV)

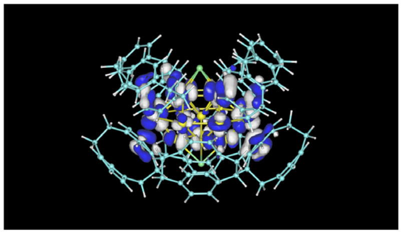

The 618th MO (-4.12 eV)

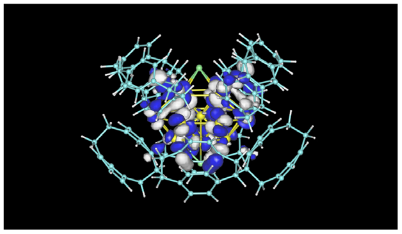

The 617th MO (-4.28 eV)
LUMO

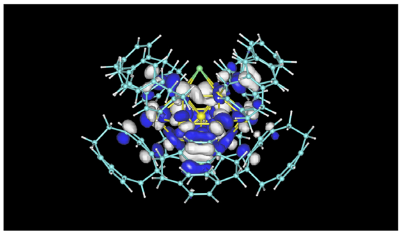

The 616th MO (-5.14 eV)
HOMO

Figure S18. The Kohn-Sham orbitals of $[Pd_{17}(\mu_3\text{-PCP})_8(\mu_4\text{-Cl})_2]^{4+}$ (**3^{4+}**).[a]

[a] Calculations were performed with the B3PW91-D3 functional using the LANL2DZ basis sets for Pd and 6-31G(d) basis sets for other atoms because clearer features of MO are generally shown by smaller basis sets than by larger basis sets with diffuse primitive functions. Numbers below figure represent the Kohn-Sham orbital energy (in eV unit).

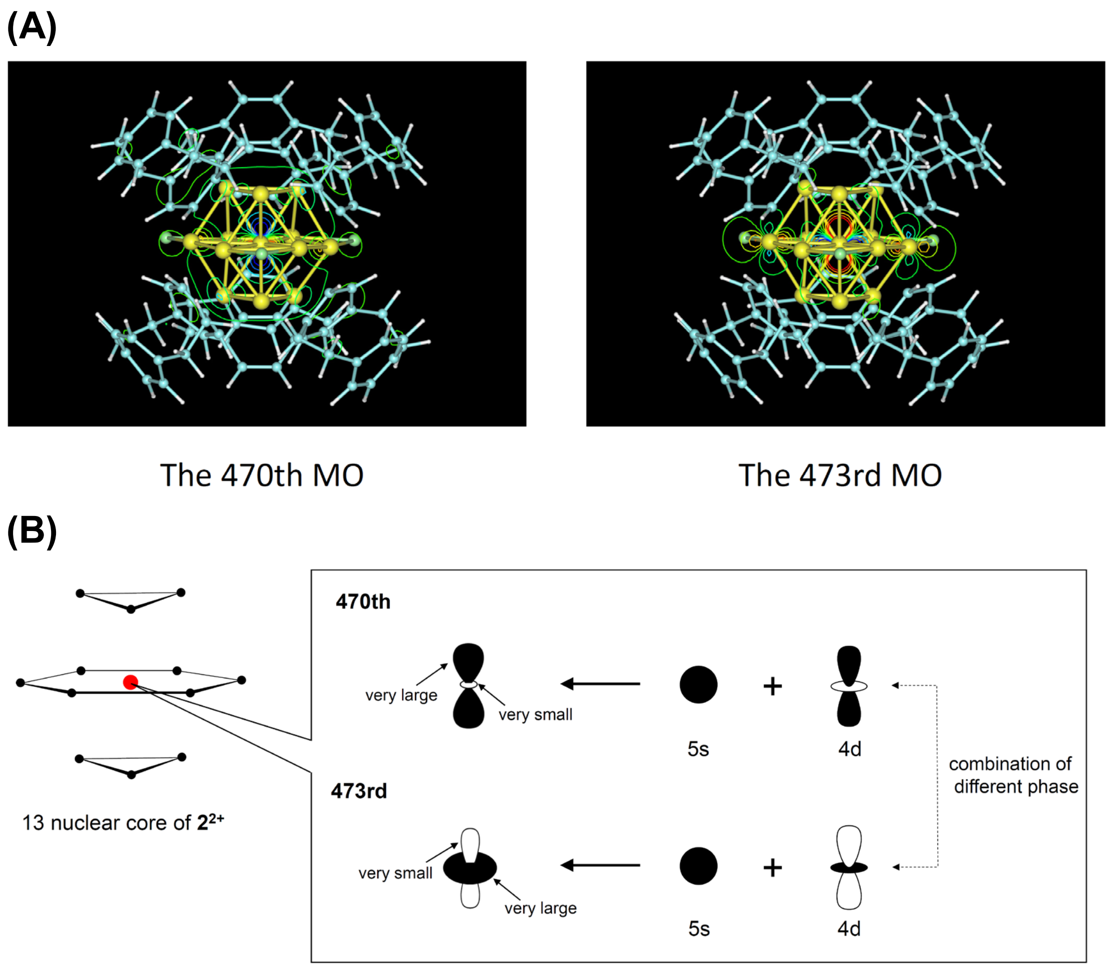

Figure S19. (**A**) The 470th and 473rd Kohn-Sham orbitals consisting of the 4d-5s hybridization on the interstitial Pd atom of $[Pd_{13}(\mu_4\text{-PCP})_6(\mu\text{-Cl})_3]^+$ (**2**$^+$).[a] (**B**) The schematic 4d-5d hybridization MOs for the interstitial Pd atom in **2**$^+$.

[a] Calculations were performed with the B3PW91-D3 functional using the LANL2DZ basis sets for Pd and 6-31G(d) basis sets for other atoms because clearer features of MO are generally shown by smaller basis sets than by larger basis sets with diffuse primitive functions.

Table S8. Summary of ΔE_{total}, ΔG^o, and Bond Energy (BE) of Gibbs Energy (kcal/mol).[a]

Reaction	ΔE_{total}	ΔG^o	BE (based on E_{total} with ZPE)
**$[Pd_{13}Cl_3(C_{16}H_{16})_6]^{2+} \rightarrow [Pd_{13}(C_{16}H_{16})_6]^{2+} + 3Cl\cdot$**	249.66	213.96	81.39 (Pd_{13}-Cl)
**$CH_2Cl\text{-}CH_2Cl \rightarrow CH_2Cl\text{-}CH_2\cdot + Cl\cdot$**	84.33	71.45	80.43 (C-Cl)
**$[Pd_{17}Cl_2(C_{16}H_{16})_8]^{3+} \rightarrow [Pd_{17}(C_{16}H_{16})_8]^{3+} + 2Cl\cdot$**	155.28	134.22	76.15 (Pd_{17}-Cl)
**$[Pd_{13}(C_{16}H_{16})_6]^{2+} + 3\ CH_2Cl\text{-}CH_2Cl \rightarrow [Pd_{13}Cl_3(C_{16}H_{16})_6]^{2+} + 3/2\ [CH_2ClCH_2\text{-}CH_2CH_2Cl]$**	-133.11	-106.35	
**$[Pd_{17}(C_{16}H_{16})_8]^{3+} + 2\ CH_2Cl\text{-}CH_2Cl \rightarrow [Pd_{17}Cl_2(C_{16}H_{16})_8]^{3+} + [CH_2ClCH_2\text{-}CH_2CH_2Cl]$**	-77.58	-62.48	

[a] Optimization were performed with the B3PW91-D3 functional using the LANL2DZ basis sets for Pd and 6-31G(d) basis sets for other atoms. The energy change of the reaction was evaluated using the solvation model based on density (SMD) method with the B3PW91-D3 functional using Stuttgart-Dresden-Bonn (SDB) basis set for Pd with corresponding ECPs and 6-311G(d) basis sets for other atoms.

Table S9. Cartesian coordinates (in Å) of the optimized geometry of $[Pd_{13}(\mu_4\text{-PCP})_6(\mu\text{-Cl})_3]^{2+}$ ($\mathbf{2}^{2+}$).

Symbol	X	Y	Z
Pd	-2.1754769	1.1512554	1.0908451
Pd	-0.0634673	-1.7820242	2.1369112
Pd	0.0650544	1.9795590	-1.9564871
Pd	-2.1741141	0.3803163	-1.5413851
Pd	0.0508375	-2.6841403	-0.7339050
Pd	-2.1826614	-1.5129598	0.4419401
Pd	2.1813822	0.3083425	-1.5482201
Pd	-0.0539242	-0.9594941	-2.6116421
Pd	0.0542181	0.7053810	2.6931152
Pd	2.1742339	-1.5062056	0.5075391
Pd	2.1788354	1.1823606	1.0519151
Pd	-0.0498265	2.7431991	0.4750811
Pd	0.0006550	0.0002239	0.0000130
Cl	0.0074432	0.9471340	-4.2511973
Cl	-0.0103518	-4.1560284	1.3068481
Cl	0.0033196	3.2086811	2.9457583
C	-2.8278817	2.4351106	-2.2588461
C	-2.5602715	3.6912087	0.3339851
C	-3.4865418	2.6298967	0.1460801
H	-4.3597788	2.6060849	0.7929951
C	1.5783271	-0.3456874	3.9478523
H	1.0485190	-0.2359163	4.8903984
C	-1.6870417	-3.3377550	-1.8410971
H	-1.3279789	-4.3617862	-1.9308001
C	-3.3611328	2.3897937	-3.6692282
H	-4.1804280	1.6638478	-3.7029752
H	-2.5787118	2.0355185	-4.3485633
C	2.8474648	3.2741026	0.4655341
C	-5.3599714	-0.2655681	4.8263154
H	-6.0982694	0.3820031	4.3578214
C	1.5981748	3.5829659	-1.6737491
H	1.0723929	4.3467680	-2.2405131
C	-3.5008995	-1.4323555	2.1883732
H	-4.3741477	-1.9781654	1.8402822
C	3.6519750	-1.3627149	-1.9163941
H	4.6074992	-0.8467500	-1.9003121
C	-1.5974294	-1.3639179	3.7123263
H	-1.0718574	-1.8664380	4.5199774
C	2.8412320	-1.2482667	-3.0673432
C	1.6831297	-2.1201916	-3.1716162

H	1.3225167	-2.2952045	-4.1840023
C	3.6458401	-0.9959978	2.1402162
H	4.6006411	-1.2438520	1.6861372
C	-3.8887066	3.7847179	-4.1812383
H	-3.1044624	4.2662618	-4.7742773
H	-4.7267737	3.5955850	-4.8611083
C	1.6936468	3.8036759	-0.2421290
H	1.3345170	4.7689870	0.1110081
C	-1.6711555	3.2687395	-1.9766761
H	-1.3072534	3.8576504	-2.8171552
C	-4.2741854	4.6792340	-3.0344392
C	3.4989196	2.0553464	-1.6192461
H	4.3698456	1.6606562	-2.1362851
C	-3.5021596	-1.9481336	6.0113125
H	-2.7684377	-2.5972518	6.4849645
C	-3.6548526	-2.0228336	-1.1884900
H	-4.6097817	-1.9640504	-0.6747780
C	-1.6888221	0.0814372	3.8118613
H	-1.3278160	0.5134322	4.7438424
C	1.6731828	-1.6959115	3.4227383
H	1.3091066	-2.4837645	4.0802683
C	-4.3789328	-2.4727965	5.0578704
C	-1.5767044	3.9057475	-0.6755550
H	-1.0470352	4.8542285	-0.6452100
C	-1.5885966	-2.5301340	-3.0435052
H	-1.0611406	-2.9807361	-3.8800092
C	2.5607783	0.6430855	3.6487993
C	-2.8410861	0.7457005	3.2256203
C	-5.3360586	4.3445662	-2.1889511
H	-6.0797087	3.6212233	-2.5170901
C	-3.6445679	2.0498217	-1.1728140
H	-4.5994510	1.5768939	-1.3824691
C	-3.6554563	0.0002776	2.3445042
H	-4.6073672	0.4214938	2.0348702
C	2.5767387	2.8239616	-2.3799461
C	-2.5673305	-1.5438747	-3.3626562
C	2.8300738	-2.0465718	2.6155352
C	-5.3900487	-1.6339892	4.5766984
H	-6.1502318	-2.0408431	3.9130513
C	-3.4922565	-1.1714285	-2.3501241
H	-4.3627004	-0.5952583	-2.6537231
C	-2.7819154	-1.1617517	-4.8060083
H	-1.8831544	-1.4311078	-5.3678163
H	-2.8884572	-0.0799346	-4.9111293

C	-2.8433008	-3.1597428	-0.9785970
C	-4.3028432	0.2968617	5.5478424
C	4.3507823	0.0901781	5.6492595
C	5.3399650	-1.8081792	4.5065044
H	6.0827651	-2.1945054	3.8116033
C	4.2815728	-2.6252801	4.9146404
C	5.3669393	-0.4635491	4.8629534
H	6.1294185	0.1874408	4.4402304
C	3.3838421	-0.6100798	-4.3221193
H	4.2032713	0.0594001	-4.0394932
H	2.6066482	0.0009354	-4.7930993
C	1.5816705	-3.2490806	-2.2645501
H	1.0510323	-4.1189226	-2.6425291
C	4.0305396	1.5572273	5.5424234
H	4.8901777	2.0906752	5.1233144
H	3.8157527	2.0133203	6.5147785
C	-3.4584143	-0.5759625	6.2426075
H	-2.6925612	-0.1673926	6.8989266
C	-4.0372316	-1.8753615	-5.4400873
H	-4.8952516	-1.1959623	-5.4028364
H	-3.8214937	-2.0579585	-6.4982394
C	3.4876598	-2.4482169	-0.9701140
H	4.3576098	-2.7033471	-0.3703980
C	3.6581657	2.3321274	-0.2055360
H	4.6109697	2.0570352	0.2368491
C	-2.8007919	-3.5686358	3.4063033
H	-1.9045519	-3.9245650	3.9221823
H	-2.9083470	-4.1995959	2.5213562
C	-3.4504002	-5.1147398	-3.6336622
H	-2.6853253	-5.8880160	-3.6031772
C	-3.9124760	1.7366298	5.3500354
H	-3.1321298	2.0069516	6.0687955
H	-4.7497969	2.4221310	5.5213004
C	2.7765645	1.7821775	4.6139384
H	1.8776435	1.8843957	5.2283854
H	2.8853557	2.7293496	4.0812553
C	3.4301849	-2.1311999	5.9088835
H	2.6622927	-2.7806878	6.3244625
C	2.5596765	-3.4863208	-1.2547130
C	3.4375965	-4.0604750	-4.7860883
H	2.6722224	-4.0921559	-5.5590174
C	-2.5797746	-2.1282178	3.0170743
C	-3.4219402	5.7101770	-2.6242331
H	-2.6512151	6.0673568	-3.3043802

C	5.3677527	-3.9968314	-2.8121542
H	6.1275397	-3.9594985	-2.0342361
C	-4.3483023	5.6408621	-0.3912140
C	3.4873893	0.3667453	2.6072602
H	4.3602655	1.0096622	2.5288302
C	-5.3654215	4.8132913	-0.8792870
H	-6.1303886	4.4487194	-0.1968050
C	3.8970996	-3.8450981	4.1221873
H	3.1152844	-4.3964700	4.6545564
H	4.7363545	-4.5368113	3.9898373
C	-3.3749059	1.9906577	3.8895613
H	-4.1895168	2.3853639	3.2730353
H	-2.5909097	2.7543186	3.9283383
C	-4.3626439	-3.1395125	-4.6898703
C	-3.4871120	-4.2286887	-4.7069123
H	-2.7491180	-4.3155619	-5.5018224
C	-5.3796350	-3.1412703	-3.7290522
H	-6.1391579	-2.3626101	-3.7544512
C	4.3770722	4.8255974	-2.9046472
C	3.3880930	4.0413286	1.6468312
H	4.2040860	3.4601844	2.0894782
H	2.6080980	4.1467227	2.4081472
C	2.7927998	3.0892796	-3.8490992
H	1.8960598	3.5753258	-4.2436843
H	2.8950616	2.1537025	-4.4031213
C	3.3655146	-3.4552570	2.6898822
H	4.1838586	-3.5500261	1.9681822
H	2.5840054	-4.1643389	2.3971042
C	4.3113453	5.5486475	-0.1863490
C	3.9159980	-1.6574209	-5.3740724
H	3.1365139	-1.8393678	-6.1210094
H	4.7590821	-1.1998451	-5.9033914
C	5.3482238	-3.0154873	-3.7981962
H	6.0941400	-2.2234754	-3.7825342
C	3.4703471	-0.7908098	6.2830955
H	2.7319261	-0.4054276	6.9833056
C	-3.4646951	6.1966310	-1.3204611
H	-2.7254859	6.9251769	-0.9935940
C	-4.0307252	5.6572271	1.0802821
H	-4.8917484	5.2855772	1.6456682
H	-3.8157080	6.6644641	1.4529431
C	-3.3826100	-4.3559508	-0.2344910
H	-4.2000360	-4.0180536	0.4113421
H	-2.6020851	-4.7711609	0.4117621

C	-2.7775313	4.7503138	1.3858261
H	-1.8784761	5.3709157	1.4364901
H	-2.8877944	4.3004848	2.3748212
C	-3.9168363	-5.4943318	-1.1863390
H	-3.1367764	-6.2530099	-1.3068160
H	-4.7572094	-5.9838166	-0.6818460
C	3.5023463	5.8204887	-2.4595331
H	2.7659054	6.2373358	-3.1436212
C	2.7686763	-4.8920230	-0.7492100
H	1.8671491	-5.4710478	-0.9681830
H	2.8761883	-4.9036210	0.3376191
C	-4.0588180	-3.7558086	4.3381774
H	-4.9170941	-4.0583945	3.7291133
H	-3.8485211	-4.5824617	5.0252084
C	4.3473754	-4.9505423	-2.7296372
C	3.9262473	5.4742226	1.2664221
H	3.1477884	6.2145328	1.4774452
H	4.7665324	5.7023024	1.9314382
C	4.2928778	-2.9556611	-4.7131303
C	5.3916292	4.4174622	-2.0319971
H	6.1503491	3.7215280	-2.3839491
C	-5.3561291	-4.0407934	-2.6678911
H	-6.0986761	-3.9575782	-1.8769191
C	4.0516060	3.9991394	-4.1201423
H	4.9078570	3.3648832	-4.3725713
H	3.8392751	4.6132315	-5.0018543
C	3.4704523	-5.0553011	-3.8127012
H	2.7288671	-5.8513910	-3.8317042
C	-4.3001902	-4.9473926	-2.5348341
C	5.3664333	4.7818173	-0.6895320
H	6.1070372	4.3696201	-0.0072670
C	4.0201593	-5.5903963	-1.4068681
H	4.8789023	-5.5005954	-0.7333300
H	3.7986390	-6.6589723	-1.4998861
C	3.4637774	6.1673857	-1.1118030
H	2.6992925	6.8560089	-0.7574560

Table S10. Cartesian coordinates (in Å) of the optimized geometry of $[Pd_{17}(\mu_3\text{-PCP})_8(\mu_4\text{-Cl})_2]^{3+}$ ($\mathbf{3}^{3+}$).

Symbol	X	Y	Z
Pd	1.8241400	-0.6028420	1.7742563
Pd	0.0083026	-0.0276117	-0.0205407
Pd	-0.5401128	-1.9521070	1.6221068
Pd	1.7135757	0.9524215	-1.7222184
Pd	-1.9430405	0.4646264	1.6618950
Pd	2.9439888	-2.1889030	-0.0451202
Pd	0.4667457	1.7699888	1.7800378
Pd	-0.9038123	1.6902569	-1.7473979
Pd	0.5055297	-3.7640421	-0.1068111
Pd	-3.7356918	-0.4614622	-0.0803716
Pd	2.2770805	2.9221313	0.1484866
Pd	-2.9904745	2.2586235	-0.1722927
Pd	3.6630548	0.5006813	0.0350370
Pd	-1.7156026	-0.9777931	-1.7512825
Pd	0.9507160	-1.7009233	-1.7695865
Pd	-0.4647938	3.6319590	0.0846468
Pd	-2.2800808	-3.0158632	-0.0901687
Cl	-0.1003854	-0.1232848	3.5265063
Cl	-0.0387560	-0.0251695	-3.5716213
H	2.6961585	4.5563753	-1.9782770
H	2.0764216	2.8417159	-3.5936911
H	0.4058683	-3.5591109	3.5363633
H	-0.5747158	3.2622746	3.7765624
H	2.7585661	5.4968349	0.8546763
H	1.8028666	2.6327201	3.9272247
H	-1.8754762	5.8986758	-0.4239312
H	0.4288628	6.1402751	0.7340822
H	-1.9792289	-2.9451069	3.6188963
H	0.6926132	-3.4414587	-3.7872999
H	-3.9801729	4.7325465	-0.6452906
H	5.2741309	0.3411559	-2.1249992
H	3.8563044	-3.5626291	2.0917910
H	3.3944746	0.4110566	3.7236481
H	-0.2017936	3.5337605	-3.6195773
H	4.2062689	-4.5627284	-0.4855118
H	-4.0422550	3.6639352	1.8632218
H	-4.6538882	-4.5797711	1.5549925
H	-4.3763616	-3.2218051	2.6434556
H	7.6888737	2.8471984	-2.5323129
H	-2.3644382	2.3775899	-3.8791468

H	5.5559731	-2.7177682	0.5260783
H	2.7567252	-2.0895500	-3.7125478
H	-2.7872822	-5.6697309	0.3782872
H	5.1124967	1.2258402	2.1985664
H	2.3057122	-5.1288940	2.3873820
H	1.6363878	-6.4758960	1.4694545
H	5.8495303	2.0517416	-0.4597417
H	7.3638915	0.4786699	3.7939946
H	-0.4479458	-6.3267967	0.3429858
H	-5.1273449	-1.1712258	2.1082954
H	-4.6010367	-4.5805362	4.4795483
H	-5.0558184	-5.8862977	3.3799759
H	-4.6726427	-4.2111437	-0.5713011
H	-0.8312424	-8.1333531	2.5711112
H	-6.3131190	0.2757004	0.5081933
H	-2.3220141	-2.5645988	-3.7859049
H	1.6153509	-7.5689367	3.4837502
H	1.7654292	-6.0829416	4.4256689
H	2.9713687	7.5949262	2.7023541
H	3.8476714	4.2101129	-5.3499349
H	5.6724173	-5.9195844	-2.6062328
H	-0.3220983	-5.4640514	-3.3926393
H	-0.2380442	-6.3434853	-1.8709372
H	3.7688335	-7.4813157	-2.3973530
H	5.6856013	-4.1785249	4.1752202
H	2.6158880	-3.3064653	4.0627717
H	2.0459551	-1.7529965	4.6808175
H	3.6026639	-3.8355748	-5.7498128
H	6.8513884	1.3221927	-4.2901726
H	2.1844034	-5.8837065	-0.5634169
H	-3.3197947	-1.3317506	4.0855643
H	2.6067074	4.6287286	5.7956105
H	-7.3252999	-0.7113251	3.8639368
H	1.7175590	-5.4223385	-5.5689293
H	7.3869760	-3.6134401	2.4723008
H	4.8991450	0.8176606	-6.0560291
H	3.5446128	1.9524284	-6.0488699
H	5.3239479	-2.6890073	-4.5939692
H	-7.8775147	-2.7967018	-2.4762044
H	6.5768170	-3.6091871	-3.7529499
H	-2.5678298	-5.5935192	-1.9244746
H	-2.2959364	-4.8194088	-3.4803684
H	-2.4876782	-4.6490408	5.6916426
H	0.1753573	5.0440659	5.8991639

H	-6.8080326	-5.0137745	-2.2672202
H	-1.8314175	7.2378343	3.8667023
H	-1.7823881	5.6985823	4.7321345
H	-2.8012848	0.2122144	4.7440849
H	6.4013483	6.0514504	-1.5243189
H	7.1840396	4.5642667	-0.9831417
H	-4.9173510	2.4859906	5.4340001
H	0.5442719	8.0372883	2.8324593
H	-8.4266873	0.8738866	2.3143096
H	-5.1702081	-1.8043206	-5.6696846
H	5.6394241	-0.0802358	5.4740489
H	-6.8535719	2.4821528	-0.3409481
H	-5.9196492	3.9173207	0.0906871
H	-3.2250592	-7.5192655	2.5752313
H	6.9723984	0.7643098	0.8191663
H	6.9851988	-0.5572821	-0.3547372
H	-3.0418027	4.5207192	-5.6831243
H	1.4371715	-7.8018907	-2.6096492
H	0.6204691	-7.4497990	-4.1366933
H	-5.3799452	-1.2623680	5.1396187
H	-4.6024366	-0.0514359	6.1638948
H	-5.9090897	-2.1439760	-0.3988606
H	2.6426379	0.3458096	-4.6241160
H	4.2209931	-0.2697777	-4.1235550
H	-2.8574958	2.2490330	3.4530514
H	-5.0010409	6.6264164	-2.4858256
H	-0.0891656	-5.2555325	5.6801182
H	-2.9188808	7.8930397	-2.0501238
H	3.8396016	-1.5055573	6.1319444
H	3.9424161	-3.2604213	5.9534679
H	0.9213122	5.3979628	-2.9901371
H	8.2638341	-1.9558917	1.0110090
H	8.7828473	-0.3960611	1.6557676
H	-5.4716743	3.1665559	-2.0262079
H	-4.7409505	2.1870778	-3.3014600
H	-6.7919417	-0.3687932	-1.5369886
H	-3.9815646	-6.3493608	-4.0145616
H	-4.7864456	-6.2338196	-2.4458517
H	-7.7393727	-0.3218630	-3.6677770
H	-5.2374418	3.7580817	-4.8735295
H	-6.1119742	4.6567872	-3.6280719
H	-6.2116425	-0.0305529	-4.5071603
H	-7.3091169	4.4199717	1.8301625
H	-8.3949283	3.1746711	1.2022616

H	-5.7860709	0.8359147	-2.3329832
H	-0.5508928	7.8882044	-2.1106121
H	0.3019544	7.5388940	-3.6180065
H	-4.1306590	-4.0371600	-5.4901015
H	-0.9683765	5.7992886	-5.2616101
H	0.8121564	6.1289529	-1.3921217
H	5.3701889	4.5918955	0.4681941
H	4.3413225	5.5180235	-0.6255955
H	4.7088300	5.7511026	-3.6175019
H	-3.5978493	-0.4566708	-3.6368401
H	4.6555176	3.7950882	2.1205692
H	-6.0058198	4.0668010	3.8730558
H	3.9823480	3.1295371	3.6052685
H	-2.4130132	4.9099622	2.6291091
H	-1.6913832	6.2699349	1.7672578
H	5.7299040	-3.1888525	-1.6271003
H	5.2184517	-1.7170943	-2.4489862
H	4.9103359	5.0997709	4.4419940
H	4.7117511	5.9981404	2.9318411
C	1.4759054	3.2044589	3.0569065
C	-2.0820445	-5.0096398	0.8878165
C	0.2311395	-4.9098575	1.8353552
C	4.6728377	0.2519717	1.9743264
C	4.5482321	1.1321142	-1.9322160
C	2.4882713	3.8926483	2.2949666
C	1.4118128	-5.7590696	2.2709072
C	2.7320128	2.5411032	-2.7743209
C	3.9353756	-2.4841353	1.9473086
C	-0.7871539	4.7389511	-1.9081402
C	4.8789885	2.1218379	-0.9517867
C	-1.8507020	4.9567252	-0.9780363
C	6.9932812	-0.5470423	3.8642732
C	3.6620666	-0.2354526	2.8855121
C	-0.6923550	-5.4004555	0.8664215
C	0.0914954	3.5697773	2.9687601
C	-4.7077542	-0.1868608	1.8935848
C	4.2396767	3.4165738	-0.9747710
C	-0.2642530	-6.6246475	4.0168433
C	-4.2465357	-5.1877186	3.6350148
C	-0.9316322	3.6255100	-2.8143088
C	0.6466917	5.1875923	1.2204783
C	-4.2249065	-3.3373897	-1.0477297
C	3.3607550	-4.1252039	-1.0213913
C	-1.1872311	-7.3578859	3.2536514

C	3.0928219	3.5540129	-1.8133425
C	-5.3986987	0.6542135	0.9646742
C	-4.1014966	2.5849082	1.7032220
C	4.7036944	-4.6755905	-4.0911106
C	3.5380313	1.3628631	-2.9349708
C	3.6289173	-3.1171510	-2.0021184
C	5.3902362	-0.6359465	1.1171952
C	6.1615077	4.3417114	-2.8485163
C	1.1868893	-4.7152086	-2.0960016
C	-5.9365465	0.7453304	4.6496530
C	-2.9159622	-2.3865105	-2.8872504
C	-1.6128643	-3.4694716	2.7355320
C	1.1889442	-6.5649006	3.6233103
C	3.3746629	-1.6368676	2.9775204
C	-4.0202121	-4.2280266	2.3794551
C	-3.2803316	-3.5427998	-2.0961486
C	2.3596515	7.0319694	3.4115824
C	2.0397741	4.7995291	1.2868031
C	4.6955293	3.9061078	-4.7311838
C	-0.3201434	4.6724089	2.1347642
C	4.8037465	-5.7996192	-3.2582831
C	-3.7931520	0.3754604	2.8621532
C	0.3753655	-5.8727217	-2.6512514
C	6.8420605	3.1529393	-3.1515770
C	1.3301544	-3.5257929	-2.9055463
C	-5.1959820	2.0769572	0.9504680
C	3.7293827	-6.6809182	-3.1401483
C	-2.1903851	2.9503698	-2.9658638
C	6.0422489	-3.1488325	4.0915145
C	-0.2247678	-3.8329403	2.6902889
C	2.9272247	-2.2743767	4.2810173
C	-3.3407393	3.3544010	-2.1997954
C	4.9142626	-1.9949252	1.0304536
C	3.6474345	-4.6416976	-5.0134316
C	-3.1153353	4.2570544	-1.1125886
C	6.3688569	2.2916644	-4.1427504
C	-2.5849157	-4.1349364	1.8990944
C	5.4268880	-2.1343492	4.8387458
C	2.1483780	-4.9135419	-1.0614754
C	2.5349173	-2.7362269	-2.8630122
C	-3.6276749	-0.2844067	4.2184340
C	5.1797562	4.7741411	-3.7522692
C	7.3837365	-1.4977139	2.9087511
C	-4.9782264	-2.1081418	-0.9690878

C	-4.8240159	-1.0711235	-1.9398604
C	7.0057395	-2.8300002	3.1320123
C	-7.6483027	1.2339310	2.9915228
C	-3.4076554	1.7416206	2.6580954
C	3.8861938	3.9571247	2.8870224
C	2.5454404	-6.4697360	-3.8634721
C	-2.1280082	-5.4125354	4.9975351
C	-7.1820864	2.5507570	2.8777522
C	-2.5371623	-7.0116610	3.2557476
C	2.9365623	5.9733058	4.1301378
C	-0.7746763	-5.7560206	4.9919239
C	5.1907889	2.5971174	-4.8388496
C	-6.4978134	-2.0884041	-3.9888776
C	5.0598456	-2.8042372	-2.4086165
C	-7.0271957	0.3401066	3.8664293
C	5.4925724	-3.4257826	-3.7947262
C	-6.4482591	-4.3122529	-3.0240292
C	-5.6815648	2.1223351	4.7428373
C	6.0203362	-0.8619991	4.8124476
C	-2.9945652	-5.9283586	4.0237078
C	3.6392357	0.6512960	-4.2741896
C	-4.1138123	6.4334302	-3.0937267
C	5.0209751	4.6553506	-0.5724390
C	-3.6678699	-1.1591395	-2.8048373
C	-6.2945254	3.0129095	3.8627980
C	-1.5037212	5.5240685	2.5521258
C	0.1622379	6.4592812	4.2657605
C	4.2008667	5.3195316	3.6304119
C	2.1611441	5.3765057	5.1349895
C	2.5808125	-5.5356928	-4.9086946
C	4.3272044	1.5076703	-5.4182056
C	-7.0509310	-3.0594129	-3.1410245
C	-3.0268898	-4.9283577	-2.6684182
C	0.7868559	5.6124895	5.1943541
C	-1.2978919	6.2780545	3.9334918
C	6.2861088	4.9583113	-1.4798089
C	0.9883534	7.2798052	3.4829724
C	-5.5406983	-2.5107584	-4.9231447
C	-6.3234687	2.9749484	0.4866896
C	6.8154935	-0.3177413	0.7066477
C	-4.1180204	5.3760471	-4.0143194
C	-4.9262654	-0.2608528	5.1340169
C	-3.0184999	5.2595236	-4.8786098
C	1.2382449	-7.0060214	-3.3415390

C	-2.9413188	7.1496218	-2.8507625
C	-5.2979956	-4.6255682	-3.7637047
C	0.2162216	5.8278701	-2.2644842
C	7.9191384	-1.0713504	1.5649887
C	4.0445722	-2.3091500	5.4110516
C	-1.7557032	6.8288706	-3.5272892
C	-6.0333163	-0.2323975	-2.3207742
C	-1.8495288	5.9823114	-4.6417342
C	-6.6802222	-0.6191371	-3.7045522
C	-4.9522340	-3.7714673	-4.8204087
C	-4.7113361	3.1712350	-2.8152084
C	-4.3131391	-5.6397193	-3.2412537
C	-5.1235830	4.2640469	-3.9049680
C	-7.3919651	3.3451494	1.6177876
C	-0.4216626	7.1305145	-2.8967475

Table S11. Cartesian coordinates (in Å) of the optimized geometry of $[Pd_{13}(\mu_4\text{-PCP})_6(\mu\text{-Cl})_3]^+$ ($\mathbf{2}^+$).

Symbol	X	Y	Z
Pd	2.1559770	-1.5672842	0.4104240
Pd	0.0722030	0.5010930	2.7537882
Pd	-0.0706131	-0.7817701	-2.6866882
Pd	2.1513161	0.4241389	-1.5709061
Pd	-0.0632729	2.7188322	0.6661781
Pd	2.1579511	1.1448790	1.1459211
Pd	-2.1581562	0.5081841	-1.5346741
Pd	0.0612630	2.1338621	-1.8114201
Pd	-0.0608472	-1.9361401	2.0216281
Pd	-2.1523212	1.0800552	1.2153621
Pd	-2.1542823	-1.5879760	0.3346830
Pd	0.0627908	-2.6357952	-0.9427541
Pd	0.0003789	0.0002740	0.0005570
Cl	-0.0108870	1.2570031	-4.1862093
Cl	0.0078671	2.9959642	3.1811022
Cl	0.0026407	-4.2535453	1.0037811
C	2.8410241	-1.0181652	-3.1627322
C	2.5449039	-3.3804984	-1.5257201
C	3.4584110	-2.3615524	-1.1436311
H	4.3294611	-2.6624484	-0.5657900
C	-1.5853423	-1.6213740	3.6372263
H	-1.0557893	-2.1732131	4.4097223
C	1.6957762	3.8330622	0.0387280
H	1.3378893	4.7665173	0.4704840
C	3.3982511	-0.2908372	-4.3616753
H	4.2124692	0.3587048	-4.0220353
H	2.6248001	0.3518959	-4.7957263
C	-2.8498734	-3.0901511	-1.1982471
C	5.3899782	-2.0930794	4.3206223
H	6.1162972	-2.4124465	3.5759673
C	-1.6001343	-2.3363891	-3.2178892
H	-1.0730553	-2.7300711	-4.0832403
C	3.4683292	0.1950138	2.6034952
H	4.3387313	0.8465358	2.5709192
C	-3.6367422	2.1061843	-0.9967161
H	-4.5920853	1.6400733	-1.2215771
C	1.6012690	-0.6496381	3.9224893
H	1.0739090	-0.6157471	4.8724904
C	-2.8516682	2.5875323	-2.0678571
C	-1.6985780	3.4080913	-1.7490411

H	-1.3422890	4.0498904	-2.5534592
C	-3.6294453	-0.1809658	2.3352062
H	-4.5853264	0.2495122	2.0498912
C	3.9456341	-1.2545883	-5.4825634
H	3.1708811	-1.3907203	-6.2444635
H	4.7879042	-0.7543313	-5.9743604
C	-1.6997584	-3.2213752	-2.0724751
H	-1.3417604	-4.2380612	-2.2278252
C	1.6873829	-1.8785912	-3.3446082
H	1.3267229	-1.9712122	-4.3679603
C	4.3295371	-2.5952764	-4.9160564
C	-3.4681114	-0.9927579	-2.4143482
H	-4.3392424	-0.3966018	-2.6769272
C	3.5426051	-1.2351523	6.1997114
H	2.8034561	-0.9098102	6.9291115
C	3.6344043	2.3330460	-0.0517790
H	4.5901044	2.0173589	0.3573350
C	1.7017419	-1.9535742	3.2941202
H	1.3443149	-2.7934923	3.8879523
C	-1.6858402	-0.1870719	3.8316483
H	-1.3249062	0.1868621	4.7886073
C	4.3984712	-0.2970303	5.6166174
C	1.5855859	-3.0749993	-2.5299352
H	1.0542408	-3.9128243	-2.9742942
C	1.5894872	3.7243662	-1.4042031
H	1.0585452	4.5286923	-1.9070701
C	-2.5463744	-2.3419120	2.8755152
C	2.8522090	-2.2258843	2.4534162
C	5.3701222	-2.7110405	-3.9902513
H	6.0999873	-1.9092975	-3.8978763
C	3.6297031	-1.2063463	-2.0059661
H	4.5848062	-0.6928213	-1.9391271
C	3.6387551	-1.1298593	2.0346331
H	4.5930612	-1.3293043	1.5549411
C	-2.5596083	-1.3145830	-3.4582642
C	2.5459043	3.0050851	-2.1727032
C	-2.8390483	0.5027222	3.2855262
C	5.4052363	-0.7777723	4.7730613
H	6.1419794	-0.0835073	4.3739863
C	3.4591513	2.1632850	-1.4828901
H	4.3282123	1.8111039	-2.0341281
C	2.7601123	3.3799571	-3.6183633
H	1.8691683	3.9089902	-3.9691773
H	2.8447203	2.4887970	-4.2440873

C	2.8496443	3.2432731	0.6906461
C	4.3528571	-2.9545824	4.6887933
C	-4.3783005	-2.8467810	4.8683034
C	-5.3686095	-0.6330548	4.7828743
H	-6.0964875	0.0445743	4.3412263
C	-4.3273544	-0.1182948	5.5601464
C	-5.3866456	-1.9798009	4.4348533
H	-6.1271937	-2.3382599	3.7227043
C	-3.4100362	2.6368304	-3.4687662
H	-4.2220193	1.9049883	-3.5410612
H	-2.6355032	2.3396003	-4.1840323
C	-1.5933380	3.9580904	-0.4105840
H	-1.0642739	4.9034714	-0.3213140
C	-4.0365326	-4.0689761	4.0582533
H	-4.8832737	-4.3261161	3.4124442
H	-3.8357896	-4.9450992	4.6850323
C	3.5144530	-2.5455814	5.7308674
H	2.7549930	-3.2305054	6.1028325
C	4.0304254	4.2887721	-3.8319553
H	4.8763635	3.6606950	-4.1320103
H	3.8248805	4.9674372	-4.6673543
C	-3.4616992	2.5955284	0.3588630
H	-4.3307383	2.5273774	1.0094691
C	-3.6373924	-1.9230790	-1.3127251
H	-4.5909225	-1.8866649	-0.7931321
C	2.7777212	1.4377600	4.7275463
H	1.8895671	1.4761000	5.3651344
H	2.8589762	2.4256260	4.2687943
C	3.4943195	6.2433793	-0.6717010
H	2.7331455	6.9045484	-0.2621530
C	3.9662420	-4.1158065	3.8125983
H	3.1957679	-4.7098995	4.3153003
H	4.8091930	-4.7895556	3.6202273
C	-2.7643955	-3.8065491	3.1649172
H	-1.8750884	-4.1895752	3.6740313
H	-2.8484985	-4.3796722	2.2389722
C	-3.4864604	-1.0348419	6.1996534
H	-2.7232753	-0.6692749	6.8838765
C	-2.5496871	3.6582294	0.5982290
C	-3.5011790	5.8774866	-2.1974112
H	-2.7403590	6.2857286	-2.8598002
C	2.5590941	0.3734979	3.6806503
C	3.4857700	-3.6988554	-5.0785434
H	2.7229199	-3.6757804	-5.8542924

C	-5.3954152	4.8258816	-0.4874810
H	-6.1338013	4.3901836	0.1825930
C	4.3746870	-4.7272606	-3.0778632
C	-3.4591054	-1.5999039	2.0787042
H	-4.3308655	-2.1262179	1.6962491
C	5.3853701	-3.7604676	-3.0772532
H	6.1257992	-3.7647396	-2.2798902
C	-3.9398803	1.3331843	5.4665974
H	-3.1642812	1.5511373	6.2083574
H	-4.7806423	2.0025124	5.6826444
C	3.4103370	-3.6274594	2.4205512
H	4.2205660	-3.6570845	1.6838211
H	2.6360559	-4.3255814	2.0846452
C	4.3771775	5.0233781	-2.5644822
C	3.5187455	5.9937163	-2.0410041
H	2.7754035	6.4592963	-2.6851232
C	5.3890545	4.5379870	-1.7297021
H	6.1275636	3.8484580	-2.1336982
C	-4.3956335	-2.7870040	-4.8858163
C	-3.4034955	-4.3295271	-0.5395190
H	-4.2156546	-4.0281351	0.1310360
H	-2.6271155	-4.7983292	0.0747360
C	-2.7817833	-0.8319569	-4.8703083
H	-1.8940643	-1.0801890	-5.4594394
H	-2.8675733	0.2564292	-4.9023254
C	-3.3929142	1.6911563	4.0322633
H	-4.2067313	2.1210204	3.4380652
H	-2.6176261	2.4586143	4.1307843
C	-4.3421426	-4.7495911	-2.8679512
C	-3.9615382	4.0561195	-3.8765603
H	-3.1892181	4.5894915	-4.4407543
H	-4.8050302	3.9057435	-4.5602503
C	-5.3828742	4.4515186	-1.8272021
H	-6.1126553	3.7300936	-2.1891572
C	-3.5173065	-2.3864820	5.8680344
H	-2.7767394	-3.0608031	6.2935275
C	3.5136639	-4.7599415	-4.1779153
H	2.7709698	-5.5513385	-4.2573393
C	4.0310289	-5.4573156	-1.8069161
H	4.8773790	-5.4000746	-1.1136701
H	3.8278989	-6.5208547	-1.9753191
C	3.4099274	3.9167571	1.9193151
H	4.2243544	3.2967400	2.3096832
H	2.6381363	3.9709631	2.6946832

C	2.7597819	-4.8197185	-1.1272331
H	1.8696368	-5.3886295	-1.4114171
H	2.8427089	-4.9164515	-0.0424470
C	3.9574955	5.3693152	1.6456681
H	3.1835505	6.0973873	1.9106581
H	4.8009985	5.5441861	2.3235142
C	-3.5347035	-3.8829091	-4.9875574
H	-2.7952105	-3.9147281	-5.7852684
C	-2.7656340	4.6420625	1.7215791
H	-1.8762919	5.2747135	1.7966521
H	-2.8486381	4.1274714	2.6813942
C	4.0521223	1.1681279	5.6155244
H	4.8963564	1.7414449	5.2171544
H	3.8518313	1.5528609	6.6218195
C	-4.3842621	5.6346986	0.0413030
C	-3.9527436	-5.3935482	-1.5642821
H	-3.1773576	-6.1449253	-1.7473271
H	-4.7924107	-5.9151462	-1.0905101
C	-4.3446761	4.8652506	-2.6665262
C	-5.4024806	-2.8443929	-3.9165843
H	-6.1428686	-2.0483278	-3.8702833
C	5.3771886	4.8041371	-0.3643850
H	6.1075836	4.3220250	0.2822450
C	-4.0553274	-1.4749839	-5.5410254
H	-4.9018415	-0.7869908	-5.4399954
H	-3.8563914	-1.5807759	-6.6133995
C	-3.5263170	6.2682436	-0.8615591
H	-2.7838519	6.9740806	-0.4944950
C	4.3390325	5.5497612	0.2009090
C	-5.3833326	-3.8189070	-2.9240482
H	-6.1102517	-3.7749920	-2.1155172
C	-4.0380181	5.5469826	1.5039181
H	-4.8833772	5.1176716	2.0527351
H	-3.8356260	6.5285787	1.9469361
C	-3.5024526	-4.8453252	-3.9823053
H	-2.7392206	-5.6206093	-4.0084773

Table S12. Cartesian coordinates (in Å) of the optimized geometry of $[Pd_{17}(\mu_3\text{-PCP})_8(\mu_4\text{-Cl})_2]^{4+}$ (**4**$^{4+}$).

Symbol	X	Y	Z
Pd	1.8647601	-0.3944210	1.7925881
Pd	0.0256080	-0.0476520	-0.0150470
Pd	-0.3257380	-1.9812761	1.6452541
Pd	1.6004761	1.1321461	-1.7045661
Pd	-1.9686851	0.2401670	1.6762541
Pd	3.1376982	-1.8042621	-0.0484220
Pd	0.2590420	1.7834031	1.7691901
Pd	-1.0565071	1.5602581	-1.7595471
Pd	0.9162361	-3.6229153	-0.1142640
Pd	-3.6362213	-0.8595991	-0.0877990
Pd	1.9065441	3.1527672	0.1564290
Pd	-3.1779402	1.8704871	-0.1772000
Pd	3.5559493	0.9145111	0.0613060
Pd	-1.5841241	-1.1301241	-1.7727431
Pd	1.1341131	-1.5665501	-1.7970431
Pd	-0.8710851	3.5048433	0.0755830
Pd	-1.8983811	-3.1961872	-0.1063980
Cl	-0.0833690	-0.1187560	3.6051003
Cl	0.0047340	-0.0125070	-3.6296943
C	2.6638722	3.8308873	-1.8236531
H	2.1309802	4.7591123	-1.9945361
C	2.4616682	2.7736922	-2.7694182
H	1.7782901	2.9607852	-3.5922863
C	0.2230680	-3.8318293	2.6897512
H	0.8234481	-3.4747912	3.5183693
C	-0.3817230	3.5096103	2.9553352
H	-1.0253551	3.0958202	3.7248073
C	1.4289301	4.9860664	1.3418561
H	2.0682261	5.7497374	0.9122671
C	1.0210151	3.3187382	3.0743532
H	1.3931761	2.7650882	3.9306883
C	-2.3342842	4.6726803	-1.0080561
H	-2.4733832	5.5934724	-0.4514670
C	0.0086780	5.1997854	1.2452441
H	-0.3163290	6.1048704	0.7417901
C	-1.1814341	-3.6284843	2.7406422
H	-1.6000931	-3.1433192	3.6153883
C	-0.9043131	4.5548483	2.1166112
C	1.7029391	-3.3423952	-2.9544762
H	1.0587531	-3.3237962	-3.8275153

C	-3.5038903	3.8258773	-1.1436461
H	-4.4124593	4.1847173	-0.6687511
C	0.7920841	-4.8331763	1.8195781
C	4.4278433	1.6369921	-1.8952141
H	5.2451704	0.9410701	-2.0461471
C	4.1805943	-2.0080551	1.9615401
H	4.2006523	-3.0848892	2.0893242
C	3.6595113	0.1864780	2.8890342
H	3.3027992	0.8017021	3.7095633
C	3.7813413	3.8445433	-0.9494741
C	-1.2733011	3.4994693	-2.8526222
H	-0.5295310	3.4710582	-3.6412583
C	3.7511483	-3.6467363	-1.0348551
H	4.6275603	-3.9807543	-0.4860830
C	-4.4074213	2.0372201	1.6790961
H	-4.4454873	3.1144282	1.8154061
C	3.5228813	-1.2272121	2.9800852
C	-3.4913053	-4.6166613	2.3391382
H	-4.0375423	-5.1141954	1.5335261
H	-3.9884403	-3.6583993	2.5173822
C	-3.8245143	-0.1070000	2.8299072
C	6.3951894	3.8596823	-3.0480692
H	7.2632565	3.6734233	-2.4194262
C	-2.4280162	2.6632932	-2.9921832
H	-2.5205232	2.0658462	-3.8949093
C	-3.6141953	2.9150792	-2.2271622
C	5.0966544	-1.4241761	1.0563861
H	5.7919934	-2.0723481	0.5351160
C	-2.0709471	-4.3772633	1.8852681
C	2.8019782	-2.4186832	-2.9094632
H	2.9355832	-1.7460521	-3.7495823
C	-1.4794711	-5.1793924	0.8787391
H	-2.1058052	-5.8850044	0.3401290
C	4.6136633	0.7740281	1.9851041
H	4.9288454	1.7921611	2.1932002
C	1.9566641	4.1277643	2.3355362
C	2.0689761	-5.5306594	2.2302302
H	2.8911232	-4.8113023	2.2992142
H	2.3363062	-6.2443145	1.4457811
C	-1.2696861	4.6035223	-1.9534821
C	4.5868213	2.6489212	-0.9067871
H	5.5354204	2.7064442	-0.3848030
C	6.9590515	0.2288560	3.8165253
H	7.2255615	1.2815171	3.7466603

C	-0.0660510	-5.4182674	0.8600631
H	0.2826250	-6.2874665	0.3108510
C	-4.6758063	-0.7643621	1.8773151
H	-4.9499194	-1.7971611	2.0670411
C	0.5096930	-6.5104815	3.9970423
C	-3.5950143	-5.4997364	3.6528543
H	-3.9489503	-4.8685264	4.4733933
H	-4.3590473	-6.2646244	3.4827933
C	-3.7663903	-3.7509753	-1.0977511
H	-4.0813853	-4.6758863	-0.6248150
C	-0.3317810	-7.3206315	3.2235342
H	0.0996060	-8.0466906	2.5378482
C	-5.4877894	-0.0100310	0.9863191
H	-6.3275785	-0.5068610	0.5154900
C	5.1831984	-4.0705153	-4.0193033
C	3.4118152	1.7093681	-2.8968842
C	3.9127233	-2.6473672	-2.0350141
C	5.4186554	-0.0286400	1.1367901
C	5.5720364	4.9578224	-2.7773452
C	1.6934841	-4.5140853	-2.1337202
C	-5.9432874	0.0188030	4.6003873
C	-2.6446612	-2.6151982	-2.9381992
H	-2.0395711	-2.6859432	-3.8369863
C	1.9482041	-6.3054744	3.6032473
H	2.4888792	-7.2519865	3.5008373
H	2.4564442	-5.7293504	4.3828733
C	-2.8366602	-3.8107403	-2.1661962
C	1.4699361	7.1457335	3.4525622
H	2.0449761	7.7879496	2.7890692
C	4.2049423	4.3275253	-4.6698133
H	3.3471852	4.5207623	-5.3105464
C	5.4017964	-5.1384654	-3.1442382
H	6.2698265	-5.1336544	-2.4882622
C	1.0191571	-5.7658744	-2.6462332
H	0.3067800	-5.4700364	-3.4177162
H	0.4463330	-6.2643775	-1.8598581
C	-5.4574084	1.4118311	0.9711951
C	4.4429733	-6.1373694	-3.0090742
H	4.5716543	-6.9069825	-2.2511332
C	6.2965945	-2.4534012	4.0495953
H	6.0598495	-3.5111043	4.1450983
C	3.1211862	-1.9089931	4.2674393
H	2.9095262	-2.9591982	4.0420073
H	2.1974472	-1.4725341	4.6585603

C	4.1415953	-4.1840073	-4.9448914
H	4.0153633	-3.4193342	-5.7082274
C	6.0553704	2.9439982	-4.0400593
H	6.6595315	2.0496231	-4.1777833
C	5.5822324	-1.5112151	4.7950173
C	2.6368922	-4.5710533	-1.0727361
H	2.7844772	-5.5166244	-0.5610020
C	-3.5528913	-0.7580001	4.1652533
H	-2.7666812	-0.1945560	4.6736723
H	-3.1537172	-1.7640451	4.0036873
C	4.5591283	5.2544384	-3.6940643
H	3.9778883	6.1672604	-3.5808213
C	7.4570605	-0.6776260	2.8739932
C	-4.6698763	-2.6357902	-0.9855991
H	-5.5651804	-2.7956262	-0.3914200
C	-4.6676183	-1.5757081	-1.9319591
C	7.2203035	-2.0376481	3.0926292
H	7.6903616	-2.7747512	2.4450072
C	-7.7157786	0.3045590	2.9689212
H	-8.4596556	-0.1382200	2.3100992
C	-3.6154723	1.2873011	2.6287592
H	-3.1155642	1.8570641	3.4070532
C	3.3368242	4.3127133	2.9245492
H	4.1022233	4.2888223	2.1457442
H	3.5353243	3.4638582	3.5870423
C	3.2660732	-6.1040675	-3.7653633
C	-1.4585061	-5.5120004	4.9939424
H	-1.8920131	-4.8069493	5.6999444
C	-7.4165065	1.6646531	2.8617622
C	-1.7058231	-7.1191795	3.2345082
H	-2.3382812	-7.6887805	2.5570522
C	2.1230512	6.1492025	4.1858663
C	-0.0799900	-5.7109034	4.9796924
H	0.5489530	-5.1568074	5.6730764
C	4.8690164	3.0992972	-4.7622283
C	-6.1598944	-2.6822432	-3.9850673
C	5.2928564	-2.1477092	-2.4027372
H	5.9836634	-2.4331182	-1.6044701
H	5.3005354	-1.0553641	-2.4497612
C	-6.9788195	-0.5115110	3.8254823
H	-7.1563365	-1.5849621	3.8288963
C	5.8232734	-2.7340592	-3.7608613
H	5.5773744	-2.0450681	-4.5757883
H	6.9155555	-2.7908002	-3.7025233

C	-5.8165894	-4.8980814	-3.0805812
H	-6.0834525	-5.6632764	-2.3548922
C	-5.8433194	1.4117061	4.6888483
H	-5.1271164	1.8571691	5.3762454
C	6.0245254	-0.1833870	4.7599123
H	5.5687554	0.5495840	5.4220174
C	-2.2679072	-6.1059504	4.0216553
C	3.5966063	0.9841231	-4.2116583
H	2.6500092	0.5494350	-4.5467243
H	4.2904673	0.1547540	-4.0447013
C	-4.7500313	5.8111084	-3.0716502
H	-5.6547994	5.8912284	-2.4729602
C	4.3797633	5.1722394	-0.5374950
H	4.7078063	5.1595184	0.5059830
H	3.5983553	5.9345884	-0.6172000
C	-3.5349223	-1.4992421	-2.8122752
H	-3.5504153	-0.7658691	-3.6115353
C	-6.5677055	2.2244052	3.8240143
H	-6.4118704	3.3010662	3.8386863
C	-2.2060742	5.2164114	2.4973002
H	-2.9900462	4.4607413	2.6052422
H	-2.5164612	5.8868294	1.6914951
C	-0.6794051	6.3374014	4.2159773
C	3.4757413	5.6532174	3.7485683
H	4.1423403	5.4631314	4.5959433
H	3.9590723	6.4061225	3.1179992
C	1.3786731	5.4506244	5.1416494
H	1.8763631	4.7537513	5.8126844
C	3.1972532	-5.2009684	-4.8292423
H	2.3459852	-5.2232974	-5.5061504
C	4.1707803	1.9071061	-5.3553944
H	4.8362893	1.3027831	-5.9805004
H	3.3438752	2.2379622	-5.9912694
C	-6.5715235	-3.7323983	-3.1581542
H	-7.4217595	-3.5980373	-2.4927192
C	-2.3840572	-5.1308504	-2.7448342
H	-1.8711311	-5.7398604	-1.9954601
H	-1.6548751	-4.9107434	-3.5279813
C	-0.0106270	5.5395504	5.1497464
H	-0.5834470	4.9094804	5.8266624
C	-2.1082732	6.0435114	3.8451743
H	-2.6909132	6.9609645	3.7141953
H	-2.5853182	5.4701044	4.6457013
C	5.6069844	5.6147874	-1.4253771

H	5.5879434	6.7075715	-1.4937951
H	6.5335515	5.3432074	-0.9103131
C	0.0824510	7.2449095	3.4715223
H	-0.4141600	7.9675606	2.8273812
C	-5.1616144	-2.9449712	-4.9272914
H	-4.8966034	-2.1824392	-5.6564744
C	-6.6549965	2.1847202	0.4816030
H	-7.1246525	1.6389251	-0.3427440
H	-6.3407815	3.1568452	0.0890050
C	6.7841555	0.4438140	0.6954801
H	6.8270495	1.5310601	0.8090821
H	6.9550945	0.2221180	-0.3643970
C	-3.5156333	4.7958293	-4.8572693
H	-3.4495362	4.0728463	-5.6672864
C	2.0300691	-6.8011395	-3.2678702
H	2.3039002	-7.5374785	-2.5056572
H	1.5065301	-7.3411305	-4.0638113
C	-4.6221853	4.7758203	-4.0012173
C	-4.8281643	-0.8578351	5.0973424
H	-5.1639214	-1.8988501	5.1320744
H	-4.5219513	-0.5843410	6.1121014
C	-3.6805493	6.6673925	-2.8242622
H	-3.7566313	7.4076035	-2.0306211
C	4.2387023	-1.8399211	5.3850464
H	3.9542093	-1.0737661	6.1124434
H	4.2496243	-2.7982132	5.9141464
C	7.9593376	-0.1934910	1.5400261
H	8.3868626	-1.0318191	0.9816761
H	8.7460576	0.5616240	1.6381421
C	-4.6542203	-5.0467544	-3.8443163
C	-0.3915490	5.7960814	-2.2662822
H	0.3676940	5.4667624	-2.9822552
H	0.1414970	6.1469864	-1.3779911
C	-2.4723842	6.5133175	-3.5109973
C	-5.9676755	-0.8695521	-2.2558922
H	-5.8528354	0.2135360	-2.1950112
H	-6.6987355	-1.1526381	-1.4929271
C	-2.4532652	5.6627834	-4.6202863
H	-1.5636031	5.6060304	-5.2438584
C	-6.5461555	-1.2644431	-3.6580133
H	-7.6313206	-1.1170581	-3.6267933
H	-6.1507144	-0.5875500	-4.4230833
C	-4.4219513	-4.1235573	-4.8676423
H	-3.5898673	-4.2702093	-5.5530194

C	-4.9460044	2.5333582	-2.8195062
H	-5.6858584	2.4378582	-2.0227621
H	-4.8429413	1.5518281	-3.2894162
C	-3.5593723	-5.9651954	-3.3741832
H	-3.1433142	-6.5776315	-4.1812533
H	-3.9563303	-6.6525285	-2.6201052
C	-5.4883344	3.5534263	-3.9094943
H	-5.5260844	3.0402442	-4.8745844
H	-6.5156535	3.8189793	-3.6429003
C	-7.7473516	2.4329232	1.6137741
H	-7.7866736	3.5055043	1.8242091
H	-8.7233086	2.1438632	1.2139281
C	-1.1879011	7.0009175	-2.8991052
H	-1.4068091	7.7353016	-2.1176372
H	-0.5361020	7.4914695	-3.6294023

Stereochemical Correlation of the Metal Cluster Core and the Ligand Shell

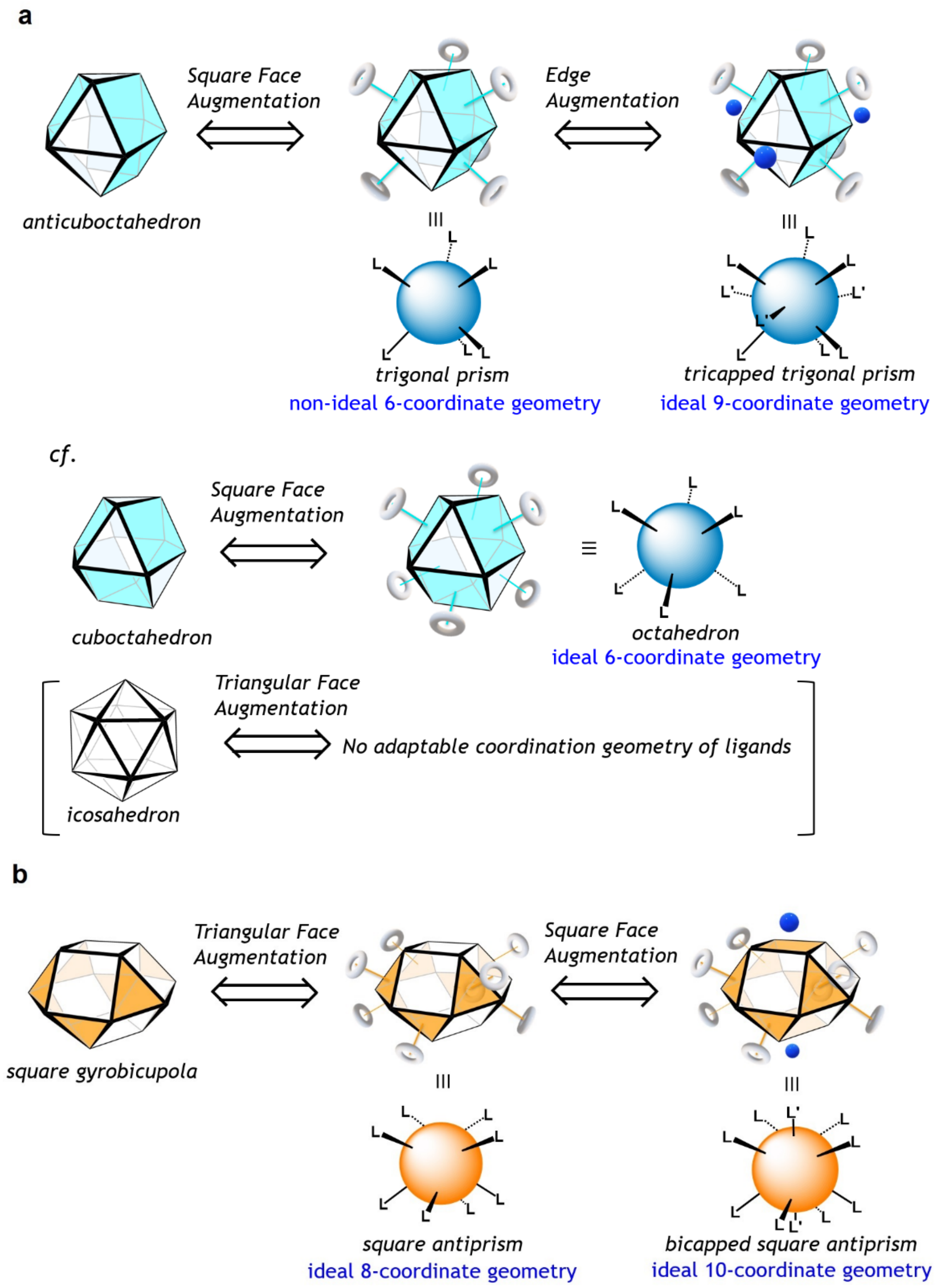

Figure S20. Stereochemical correlation of the metal cluster core and the ligand shell. **a**, Anticuboctahedron ⇔ trigonal prism ⇔ tricapped trigonal prism. **b**, Square gyrobicupola ⇔ square antiprism ⇔ bicapped square antiprism.

References

1. Murahashi, T.; Fujimoto, M.; Kawabata, Y.; Inoue, R.; Ogoshi, S.; Kurosawa, H. Discrete Triangular Tripalladium Sandwich Complexes of Arenes. *Angew. Chem. Int. Ed.* **2007**, *46*, 5440-5443.
2. Yakelis, N. A.; Bergman, R. G. Safe Preparation and Purification of Sodium Tetrakis[(3,5-trifluoromethyl)phenyl]borate ($NaBArF_{24}$): Reliable and Sensitive Analysis of Water in Solutions of Fluorinated Tetraarylborates. *Organometallics* **2005**, *24*, 3579-3581.
3. Murahashi, T.; Nagai, T.; Okuno, T.; Matsutani, T.; Kurosawa, H. Synthesis and ligand substitution reactions of a homoleptic acetonitrile dipalladium(I) complex. *Chem. Commun.* **2000**, 1689-1690.
4. Sheldrick, G. M. SHELXT – Integrated space-group and crystal-structure determination. *Acta Crystallogr.* **2015**, *A71*, 3-8.
5. Sheldrick, G. M. Crystal structure refinement with SHELXL. *Acta Crystallogr.* **2015**, *C71*, 3-8.
6. Farrugia, L. J. WinGX and ORTEP for Windows: an update. *J. Appl. Crystallogr.* **2012**, *45*, 849-854.
7. Becke, A. D. Density-functional Exchange-energy Approximation with Correct Asymptotic Behavior. *Phys. Rev. A* **1988**, *38*, 3098-3100.
8. Becke, A. D. Density-functional Thermochemistry. III. The Role of Exact Exchange. *J. Chem. Phys.* **1993**, *98*, 5648-5652.
9. Perdew, J. P.; Chevary, J. A.; Vosko, S. H.; Jackson, K. A.; Pederson, M. R.; Singh, D. J.; Fiolhais, C.; Atoms, Molecules, Solids, and Surfaces: Applications of the Generalized Gradient Approximation for Exchange and Correlation. *Phys. Rev. B: Condens. Matter Mater. Phys.* **1992**, *46*, 6671-6687.
10. Perdew, J. P.; Chevary, J. A.; Vosko, S. H.; Jackson, K. A.; Pederson, M. R.; Singh, D. J.; Fiolhais, C. Erratum: Atoms, Molecules, Solids, and Surfaces: Applications of the Generalized Gradient Approximation for Exchange and Correlation. *Phys. Rev. B: Condens. Matter Mater. Phys.* **1993**, *48*, 4978-4990.

11. Perdew, J. P.; Burke, K.; Wang, Y. Generalized Gradient Approximation for the Exchange-correlation Hole of A Many-electron System. *Phys. Rev. B: Condens. Matter Mater. Phys.* **1996**, *54*, 16533-16539.
12. Grimme, S.; Antony, J. S.; Ehrlich, S.; Krieg, H. A Consistent and Accurate Ab Initio Parametrization of Density Functional Dispersion Correction (DFT-D) for the 94 Elements H-Pu. *J. Chem. Phys.* **2010**, *132*, 154104.
13. Ryde, U.; Matab, R. A.; Grimme, A. Does DFT-D Estimate Accurate Energies for the Binding of Ligands to Metal Complexes? *Dalton Trans.* **2011**, *40*, 11176-11183.
14. Becke, A. D.; Johnson, E. R. A Density-Functional Model of the Dispersion Interaction. *J. Chem. Phys.* **2005**, *123*, 154101−154106.
15. Becke, A. D.; Johnson, E. R. Exchange-Hole Dipole Moment and the Dispersion Interaction. *J. Chem. Phys.* **2005**, *122*, 154104−154109.
16. Johnson, E. R.; Becke, A. D. A Post-Hartree-Fock Model of Intermolecular Interactions: Inclusion of Higher-Order Corrections. *J. Chem. Phys.* **2006**, *124*, 174104−174112 (2006).
17. Hay, P. J.; Wadt, W. R. Ab initio Effective Core Potentials for Molecular Calculations. Potentials for K to Au Including the Outermost Core Orbitals. *J. Chem. Phys.* **1985**, *82*, 299-310.
18. Andrae, D.; Häußermann, U.; Dolg, M.; Stoll, H.; Preuß, H. Energy-adjustedab initio Pseudopotentials for the Second and Third Row Transition Elements: Molecular Test for M2 (M = Ag, Au) and MH (M=Ru, Os). *Theor. Chim. Acta* **1991**, *78*, 247-266.
19. Reed, A. E.; Curtiss, L. A.; Weinhold, F. Intermolecular interactions from a natural bond orbital, donor-acceptor viewpoint. *Chem. Rev.* **1988**, *88*, 899-926.
20. Hirshfeld, F. L., Bonded-atom fragments for describing molecular charge densities. *Theor. Chem. Acc.* **1977**, *44*, 129-138.
21. Marenich, A. V.; Cramer, C. J.; Truhlar, D. G. Universal solvation model based on solute electron density and a continuum model of the solvent defined by the bulk dielectric constant and atomic surface tensions. *J. Phys. Chem. B*, **2009**, *113*, 6378-6396.
22. Frisch, M. J.; Trucks, G. W.; Schlegel, H. B.; Scuseria, G. E.; Robb, M. A.; Cheeseman, J. R.; Scalmani, G.; Barone, V.; Mennucci, B.; Petersson, G. A. *et al.* Gaussian 16, Revision C.01, Gaussian, Inc.: Wallingford CT, 2016.
